# The impact of an ICME on the Jovian X‐ray aurora

**DOI:** 10.1002/2015JA021888

**Published:** 2016-03-22

**Authors:** William R. Dunn, Graziella Branduardi‐Raymont, Ronald F. Elsner, Marissa F. Vogt, Laurent Lamy, Peter G. Ford, Andrew J. Coates, G. Randall Gladstone, Caitriona M. Jackman, Jonathan D. Nichols, I. Jonathan Rae, Ali Varsani, Tomoki Kimura, Kenneth C. Hansen, Jamie M. Jasinski

**Affiliations:** ^1^ Mullard Space Science Laboratory, Department of Space and Climate PhysicsUniversity College LondonDorkingUK; ^2^ Centre for Planetary ScienceUCL/BirkbeckLondonUK; ^3^ ZP12, NASA Marshall Space Flight CenterHuntsvilleAlabamaUSA; ^4^ Center for Space PhysicsBoston UniversityBostonMassachusettsUSA; ^5^ LESIA, Observatoire de Paris, CNRS, UPMCUniversité Paris DiderotMeudonFrance; ^6^ Kavli Institute for Astrophysics and Space ResearchMITCambridgeMassachusettsUSA; ^7^ Space Science and Engineering DivisionSouthwest Research InstituteSan AntonioTexasUSA; ^8^ Department of Physics and AstronomyUniversity of SouthamptonSouthamptonUK; ^9^ Department of Physics and AstronomyUniversity of LeicesterLeicesterUK; ^10^ Space Research InstituteAustrian Academy of SciencesGrazAustria; ^11^ Institute of Space and Astronautical ScienceJapan Aerospace Exploration AgencySagamiharaJapan; ^12^ Nishina Center for Accelerator‐Based ScienceRIKENWakoJapan; ^13^ Department of Atmospheric, Oceanic and Space SciencesUniversity of MichiganAnn ArborMichiganUSA

**Keywords:** Jupiter, X‐ray, Aurora, CME, Periodicity, Jovian

## Abstract

We report the first Jupiter X‐ray observations planned to coincide with an interplanetary coronal mass ejection (ICME). At the predicted ICME arrival time, we observed a factor of ∼8 enhancement in Jupiter's X‐ray aurora. Within 1.5 h of this enhancement, intense bursts of non‐Io decametric radio emission occurred. Spatial, spectral, and temporal characteristics also varied between ICME arrival and another X‐ray observation two days later. Gladstone et al. (2002) discovered the polar X‐ray hot spot and found it pulsed with 45 min quasiperiodicity. During the ICME arrival, the hot spot expanded and exhibited two periods: 26 min periodicity from sulfur ions and 12 min periodicity from a mixture of carbon/sulfur and oxygen ions. After the ICME, the dominant period became 42 min. By comparing Vogt et al. (2011) Jovian mapping models with spectral analysis, we found that during ICME arrival at least two distinct ion populations, from Jupiter's dayside, produced the X‐ray aurora. Auroras mapping to magnetospheric field lines between 50 and 70 *R*_*J*_ were dominated by emission from precipitating sulfur ions (S^7+,…,14+^). Emissions mapping to closed field lines between 70 and 120 *R*_*J*_ and to open field lines were generated by a mixture of precipitating oxygen (O^7+,8+^) and sulfur/carbon ions, possibly implying some solar wind precipitation. We suggest that the best explanation for the X‐ray hot spot is pulsed dayside reconnection perturbing magnetospheric downward currents, as proposed by Bunce et al. (2004). The auroral enhancement has different spectral, spatial, and temporal characteristics to the hot spot. By analyzing these characteristics and coincident radio emissions, we propose that the enhancement is driven directly by the ICME through Jovian magnetosphere compression and/or a large‐scale dayside reconnection event.

## Key Points

- The arrival of an ICME changes Jupiter's X‐ray auroral spectra, spatial, and temporal characteristics
- Jupiter's X‐ray aurora maps to sources in the outer magnetosphere and also to open field lines
- Jupiter's X‐ray aurora is produced by two distinct ion populations during the ICME

## 1 Introduction

The Einstein Observatory first permitted the identification of Jupiter's X‐ray emission during the 1980s [*Metzger et al.*, 1983]. Since then, Röntgen satellite, Chandra, and XMM‐Newton X‐ray observatories have provided the opportunity to study the spatial, spectral, and temporal characteristics of this X‐ray emission in more detail [*Waite et al.*, 1994; *Gladstone et al.*, 1998, 2002; *Elsner et al.*, 2005; *Branduardi‐Raymont et al.*, 2004, 2007a, 2007b, 2008; *Bhardwaj et al.*, 2005, 2006]. Jupiter's X‐ray emission consists of two components: an equatorial/disk component and a high‐latitude north and south auroral component [*Metzger et al.*, 1983; *Waite et al.*, 1994]. The disk emission is found to be dominated by elastic and fluorescent scattering of solar X‐ray photons in the upper atmosphere, meaning that changes in the Sun's X‐ray emission induce changes in Jupiter's disk emission [*Maurellis et al.*, 2000; *Branduardi‐Raymont et al.*, 2007b; *Bhardwaj et al.*, 2005, 2006; *Cravens et al.*, 2006]. The majority of the auroral X‐ray emission above ∼60° latitude is thought to be due to charge exchange (CX) interactions between precipitating ions and atmospheric neutral hydrogen molecules [*Waite et al.*, 1994; *Cravens et al.*, 1995, 2003; *Cravens and Ozak*, 2012]. The origin of the ions, however, has been a matter of debate; they could either come from the magnetosphere or from the solar wind. In this work we explore the question of ion origin and, in particular, we analyze changes to the Jovian X‐ray emission during heightened solar wind conditions to better understand the factors driving the emission.

Jupiter's northern X‐ray aurora is dominated by two distinctive spectral emissions, which are each associated with distinct spatial emissions: (1) the hot spot region containing ion‐produced CX soft X‐ray line emissions (0.2–2 keV) [*Gladstone et al.*, 2002] and (2) the electron‐produced hard X‐ray (greater than 2 keV) bremsstrahlung continuum emission, which appears to overlap the UV main oval region [*Branduardi‐Raymont et al.*, 2008].

### 1.1 Soft X‐Rays and the Hot Spot Region

Poleward of the main auroral oval, and therefore magnetically mapping to larger radial distances, is the “hot spot” region discovered by *Gladstone et al.* [2002]. This region is found to be the dominant X‐ray feature in Jupiter's northern aurora and appears to be roughly fixed in System III (S3) coordinates of 160°–180° longitude and 60°–70° latitude [*Gladstone et al.*, 2002]. Using the VIP4 model [*Connerney et al.*, 1998], *Gladstone et al.* [2002] mapped the hot spot to magnetospheric origins farther than 30 *R*_*J*_ from the planet. Poleward of regions mapping to 30 *R*_*J*_ the VIP4 model does not permit accurate mapping of the ionosphere to the magnetosphere [*Vogt et al.*, 2011], so the precise magnetospheric origin of the hot spot remained unknown.

Pertinent to understanding the Jovian magnetosphere is the 45 min periodicity that *Gladstone et al.* [2002] also detected in the X‐ray hot spot. *Elsner et al.* [2005] and *Branduardi‐Raymont et al.* [2004, 2007b] were unable to find strict periodicity in Chandra or XMM‐Newton observations in 2003 and 2004 but noted that periodic behavior may be transient and linked to solar activity.

Chandra and XMM‐Newton observations have shown that the hot spot emission is dominated by soft X‐ray CX spectral lines from ions [*Elsner et al.*, 2005; *Branduardi‐Raymont et al.*, 2004, 2007b]. Further, these authors showed that dominant constituents of this emission are fully stripped and almost fully stripped oxygen ions, whose CX emission (characterized by strong O VII and O VIII line emission) fits well to the observed 500–900 eV spectra. The authors also showed that at lower energies, between 200 and 400 eV, there are likely to be CX lines from carbon or sulfur ions, but spectral resolution has been insufficient to distinguish between these species.

Determining whether the low‐energy lines are due to carbon or sulfur ions is fundamental to determining whether Jupiter's magnetosphere is open or closed to the solar wind. Carbon and oxygen are the most abundant heavy ions in the solar wind [*Cravens*, 1997], so a carbon confirmation would suggest a solar wind origin for the emission. Conversely, Jupiter's magnetosphere is dominated by sulfur and oxygen ions, which are produced by Io's volcanoes and diffuse to the outer magnetosphere. Sulfur identification would indicate that these precipitating ions are magnetospheric in origin. While the Jovian magnetosphere is dominated by sulfur and oxygen, these are predominantly in charge states of S^+^, S^2+^, and S^3+^ or O^+^ and O^2+^ [*Geiss et al.*, 1992]. For X‐ray production by CX, charge states of at least S^7+^ and O^7+^ are required, so the ions require acceleration to enable collisions to strip electrons and generate the higher charge states observed.

*Cravens et al.* [2003] proposed two mechanisms capable of explaining the CX emission; one for ions originating in the solar wind and the other for ions originating in the magnetosphere. If the ions are carbon, then they would already have the required charge state in the solar wind. However, *Cravens et al.* [2003] show that under normal solar wind conditions, without an acceleration process, the low densities of solar wind ions at Jupiter are only capable of explaining 0.5–5% of the observed emission. A field‐aligned potential drop of ∼200 kV between the magnetopause and upper atmosphere is required to accelerate the particles to sufficient energies to generate the emission from cusp precipitation alone. At these energies, bright UV emission should be observable from precipitating protons, but this is only observed during short‐lived flare events [*Trafton et al.*, 1998; *Waite et al.*, 2001]. These bright polar cap UV flares were attributed to the cusp by *Pallier and Prangé* [2001]. Outside of flares, *Cravens et al.* [2003] found that cusp precipitation could only be partially responsible for the emission. Instead, they favored a mechanism where field‐aligned electric potentials (of at least 8 MV) in the outer magnetosphere accelerate local sulfur and oxygen ions to the required energies. The location of downward currents in this region is also supported by work of *Nichols* [2011].

*Bunce et al.* [2004] proposed an alternative scenario. They suggested that pulsed dayside reconnection at the magnetopause could generate the observed X‐ray emission. They showed that this would lead to the precipitation of solar wind ions, but that it actually drives more intense X‐ray emission from closed outer magnetosphere field lines that are perturbed by reconnection flows. This would mean that the greater emission intensity would be from sulfur in the outer magnetosphere. The authors also indicated that pulsed reconnection could explain the period observed by *Gladstone et al.* [2002], suggesting that a 30–50 min period would be expected from this process. *Bonfond et al.* [2011] suggest that the quasiperiodic UV flaring with timescales of 2–3 min found poleward of the main oval, in a region close to the X‐ray hot spot, may also be caused by pulsed dayside reconnection.

When investigating the Jovian X‐ray aurora spectra, *Branduardi‐Raymont et al.* [2004, 2007b] and *Elsner et al.* [2005] showed a slight preference for sulfur and therefore a magnetospheric origin, but *Elsner et al.* [2005] concluded that they were unable to rule out carbon. Further modeling [*Hui et al.*, 2009, 2010; *Kharchenko et al.*, 2006, 2008; *Ozak et al.*, 2010, 2013] has demonstrated that a good fit to the spectra can be found with a combination of 1–2 MeV/amu oxygen and sulfur ion lines. *Hui et al.* [2010] also found that the majority of spectra could be well fitted without carbon lines, although one set of spectra had a better fit with a carbon‐oxygen model. They also noted significant variation between observation dates and between northern and southern auroras. This north‐south pole variation may be expected because Jupiter's 9.6° dipole tilt ensures that the viewing geometry of one pole is always significantly impaired relative to the other. This means that additional spatial features (and the spectral lines associated with them) can be viewed more clearly for one pole than the other. Additionally, the magnetic field footprints in the north pole feature a significant kink structure between 90° and 150° S3 longitude [*Pallier and Prangé*, 2001], which is well fitted by a magnetic anomaly [*Grodent et al.*, 2008]; this is absent from the south pole, which may relate to its more diffuse X‐ray emission [*Elsner et al.*, 2005].

### 1.2 Hard X‐Rays and the Main Oval

Equatorward of the hot spot is the UV main oval. By comparing Chandra auroral X‐ray events, *Branduardi‐Raymont et al.* [2008] showed that hard X‐rays (energies greater than 2 keV) map well to the main UV oval. This emission is found to be well fitted by bremsstrahlung radiation from precipitating electrons [*Branduardi‐Raymont et al.*, 2007b], implying a spatial coincidence of the X‐ray and UV‐emitting electron populations.

The main oval is well evidenced as mapping to 20–30 *R*_*J*_ [*Vogt et al.*, 2011], where upward field‐aligned currents in the corotation breakdown region could generate downward precipitation of 20–100 keV electrons [*Hill*, 2001; *Cowley and Bunce*, 2001; *Nichols and Cowley*, 2004]. This region is significantly separated from the 63–92 *R*_*J*_ standoff distance calculated by *Joy et al.* [2002], and thus, emission might not be expected to be directly influenced by the solar wind. However, in contrast with this apparent isolation, *Branduardi‐Raymont et al.* [2007b] note that in 2003 XMM‐Newton observations showed that both hard and soft X‐ray emissions varied at a time of increased solar activity [*Branduardi‐Raymont et al.*, 2004, 2007b]. UV main emissions connected with the hard X‐ray emission are also known to be modulated by the solar wind [*Pryor et al.*, 2005; *Nichols et al.*, 2007; *Clarke et al.*, 2009; *Nichols et al.*, 2009].

### 1.3 Connecting Solar Wind and Auroral Variations

While the impact of a southward turning interplanetary magnetic field and the pressure pulse induced by an interplanetary coronal mass ejection (ICME) on the Earth's aurora are known to produce auroral brightening [*Elphinstone et al.*, 1996; *Chua et al.*, 2001], the impact on Jupiter's larger magnetosphere is not well understood. There are two key challenges associated with examining relationships between solar wind conditions and the Jovian aurora. First, the timescales for the propagation of a solar wind‐induced shock through the Jovian magnetosphere are not well understood. Second, without in situ measurements of the solar wind conditions close to Jupiter, we rely on propagation models to estimate the solar wind conditions upstream of Jupiter. The propagation of the solar wind beyond the inner heliosphere becomes increasingly complex, meaning that outside of certain limiting conditions (e.g., Jupiter in opposition) the uncertainty associated with propagation models can be on the order of days, making it difficult to precisely correlate solar activity with auroral intensification. However, *Gurnett et al.* [2002] found that Jovian hectometric radio emission bursts (0.3–3 MHz) coincided with maxima in solar wind density. *Prangé et al.* [2004] and *Lamy et al.* [2012] have used these enhancements in radio emission to trace the progress of ICME‐induced shocks through the solar system. Further, *Echer et al.* [2010] and *Hess et al.* [2012, 2014] found that non‐Io decametric radio emission bursts are correlated with periods of increased solar wind dynamic pressure.

Jupiter's auroral variations in response to changes in solar wind pressure are well catalogued at other wavelengths [*Barrow et al.*, 1986; *Ladreiter and Leblanc*, 1989; *Kaiser*, 1993; *Prangé et al.*, 1993; *Baron et al.*, 1996; *Zarka*, 1998; *Pryor et al.*, 2005; *Nichols et al.*, 2007; *Clarke et al.*, 2009; *Nichols et al.*, 2009; *Hess et al.*, 2012, 2014], but X‐ray emission is yet to be investigated in this manner. In particular, there have been few previous opportunities to connect X‐ray observations of high‐latitude precipitating ions with solar wind conditions. There has also been limited analysis of how the spatial morphology of X‐ray features varies over time. In the current work, we analyze auroral spatial features, connect them with spectral features, and compare their morphology and evolution over time to better understand how solar wind conditions and local time magnetosphere variation might drive them.

In section 2, we consider the propagation of an ICME to Jupiter and describe how two Chandra X‐ray observations and radio measurements were timed to coincide with the expected arrival time of the ICME at the planet. In section 3, we present polar projections of the X‐ray events, identifying changes in their spatial distribution between the observations. In section 4, we compare the auroral lightcurves for each observation and find connections between a bright X‐ray auroral enhancement and decametric radio emission thought to be induced by the ICME. In section 5, we compare the spectra for the hot spot and the auroral enhancement, identifying changes between the observations, which are possibly induced by the ICME. We then compare the X‐ray polar projections for specific energy ranges (section 6), based on the different precipitating particles species generating the emission. For instance, we provide polar projections for X‐ray emission only from oxygen ions, in order to compare this with other ion species and electrons. By doing this, we find that there is an X‐ray auroral region closer to the UV main oval that is dominated by emission from high charge‐state ions of sulfur or carbon. While poleward of this, the population is more of a mixture of high charge‐state oxygen and high charge‐state carbon/sulfur ions. In section 7, we bin the X‐ray events based on the timing of specific subsolar longitudes (noon times) and use these to identify how auroral developments relate to the evolution of the magnetosphere. Using the *Vogt et al.* [2011] model, we map the magnetospheric source and local time dependencies of the hot spot region and the auroral enhancement region. This indicates to what extent X‐ray emission may be driven by the opening/closing of magnetic field lines, the location of the Sun relative to Jupiter's magnetosphere, and the magnetosphere's auroral footprints. In section 8, we investigate periodicities in the X‐ray emission and their relationships to specific ion species. In sections 9–11 we summarize results, provide discussion on these, and draw conclusions.

## 2 October 2011 Jupiter Observations

The two Chandra X‐ray observations reported here were undertaken to attempt to establish if and to what extent the solar wind drives Jupiter's X‐ray aurora. Having previously observed variations in X‐ray emission possibly associated with increased solar activity [*Branduardi‐Raymont et al.*, 2007b], an extreme solar event such as an ICME was thought to provide the opportunity to better understand this connection. To minimize the uncertainty associated with models that propagate the solar wind conditions to Jupiter and to maximize the X‐ray flux and spatial resolution, it is important that Jupiter is observed close to opposition, with the smallest possible Earth‐Sun‐Jupiter angle. Opposition occurred in October 2011, so a Chandra Target of Opportunity (TOO) proposal was submitted to observe Jupiter at the time when an ICME was predicted to arrive.

**Figure 1:**
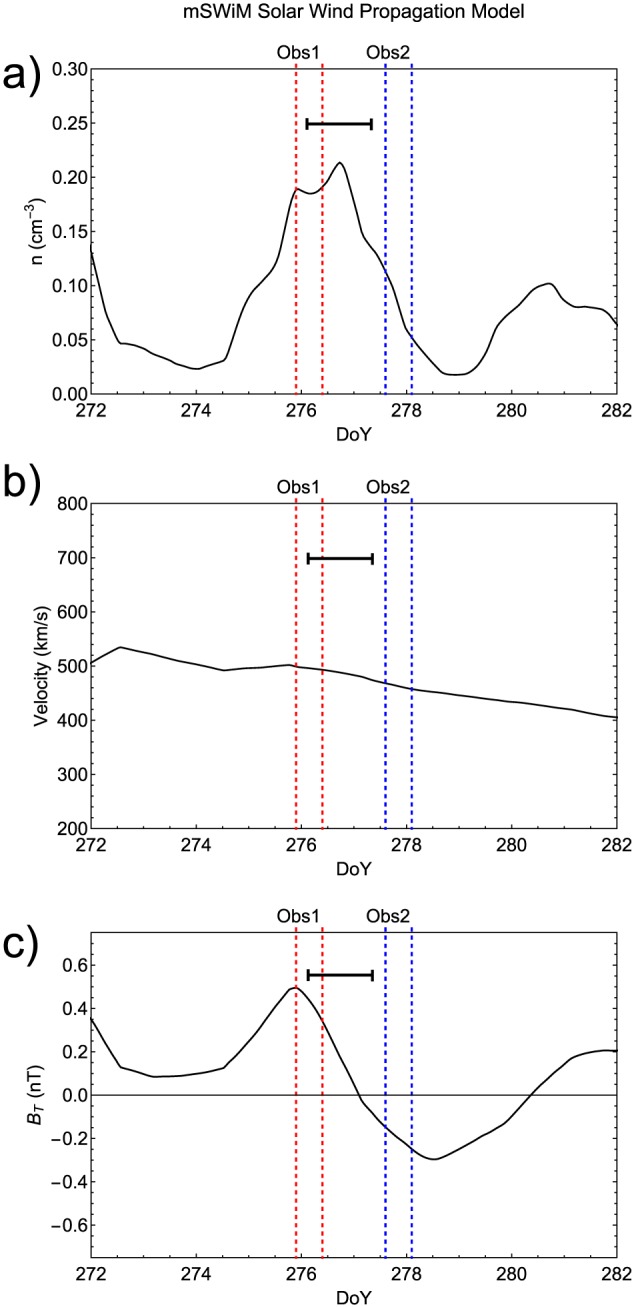
mSWiM propagation model [*Zieger and Hansen*, 2008] at Jupiter on a given day of year in 2011. (a) Solar wind density, (b) velocity, and the (c) *B*_*T*_ magnetic field component. Start/end times of Chandra X‐ray observations are shown by dashed lines for the first (red) and second (blue) observations (see text for details). The 10–15 h uncertainty in the model is indicated by the black bar toward the top of each parameter plot.

We used the 1.5‐D MHD mSWiM model [*Zieger and Hansen*, 2008; http://mswim.engin.umich.edu/] to determine the solar wind parameters at Jupiter. This allowed us to propagate solar wind measurements from 1 AU to Jupiter. Inspection of the solar wind density, velocity, and the interplanetary magnetic field (IMF) timelines (Figure 1) indicated the predicted arrival of an ICME at Jupiter over 2 and 3 October 2011, day of year (DOY) 275–276 (Figure 1). At this time, the Earth‐Sun‐Jupiter angle was ∼25° and Jupiter was ∼4.07 AU from the Earth, meaning that the propagation model offered a relatively low uncertainty of 10–15 h and that Jupiter was within the angular extent of the ICME [*Robbrecht et al.*, 2009a, 2009b]. To account for this uncertainty, we smooth the mSWiM propagations over a 30 h moving average.

The most accurate parameter is solar wind velocity, followed by density and the tangential component of the magnetic field (*B*_*T*_) [*Zieger and Hansen*, 2008], which points toward the cross product of the solar rotation vector and the direction radially away from the Sun toward Jupiter. Inspecting the mSWiM model propagations of the solar wind reveals an increase in density from 0.03 cm^−3^ on DOY 274.5 to a peak of 0.21 cm^−3^ on DOY 276.75 (Figure 1a). Density then decreases from this peak back to a minimum of 0.015 cm^−3^ on DOY 279.0. The median densities measured upstream of Jupiter by Pioneer 11, Voyager 1, and Voyager 2 were 0.13, 0.14, and 0.15 cm^−3^, respectively, indicating that the mSWiM averaged solar wind density is above the median value [*Jackman and Arridge*, 2011] (see supporting information for these distributions). There is also a modest increase in solar wind velocity during this time from 490 km/s on DOY 274.5 to 500 km/s on DOY 276.0 (Figure 1b). This then decreases gradually to 450 km/s by DOY 279.0. These solar wind velocities are similar to the median velocity upstream of Jupiter measured by Pioneer 11 (493 km/s) but represent an increase over the Voyager 1 and 2 median velocities (439 and 441 km/s, respectively). The mSWiM‐predicted density and velocity is much closer to the mean from Pioneer 11, Voyager 1, and Voyager 2 upstream measurements (0.26, 0.23, and 0.25 cm^−3^ and 497, 446, and 448 km/s respectively ), suggesting that the variation in solar wind conditions represent a more modest ICME.

The *B*_*T*_ magnetic field plot appears to show a rotation in the solar wind magnetic field at this time, with the field oriented in the positive *B*_*T*_ direction from DOY 274.5 to DOY 277 and a negative *B*_*T*_ direction from DOY 277 to 280, before returning to a positive orientation again (Figure 1c). This variation in IMF along with the simultaneous increase in density and velocity is consistent with an ICME with flux rope‐like interior structure [*Hanlon et al.*, 2004].

We also note that the mSWiM model shows that a much stronger ICME was incident at Jupiter from DOY 268 to 272 and the solar wind can be seen to be returning to non‐ICME conditions from DOY 272.5. The arrival of this preceding ICME is also accompanied by bursts of Jovian radio emission [*Lamy et al.*, 2012]. It is possible that this preceding ICME may also have driven changes in the Jovian magnetosphere, which are still observable in the X‐ray observations reported here.

### 2.1 Chandra X‐Ray Observations

Based on the predicted arrival of the ICME at Jupiter, two TOO observations were made by the Chandra X‐ray Observatory Advanced CCD Imaging Spectrometer (ACIS). Each observation lasted 11 h, providing coverage of at least one full Jupiter rotation (∼9 h 55 min). Two observations separated by a couple of days were requested in order to optimize our chances to observe Jupiter during the ICME impact and during relaxed conditions. Both observations were made with the back‐illuminated (S3) CCD, which has the highest sensitivity to low‐energy X‐rays. To simplify the analysis, the observatory was oriented so that the moving image of Jupiter remained on the same output node of the CCD throughout each observation. The first observation was timed to coincide with the predicted arrival of the ICME at Jupiter and lasted from ∼ 21:55 on 2 October to 09:30 on 3 October 2011 (day of year 275.9–276.4). The second observation ran from 14:35 on 4 October until 02:20 on 5 October 2011 (day of year 277.6–278.1). Figure 1 shows the times of these observations between red (first observation) and blue (second observation) dotted lines plotted onto the mSWiM solar wind propagation diagram. These suggest that the density peak occurred toward the end of the observation. The second observation occurred when solar wind density was returning to conditions outside of an ICME‐induced shock. Figure 1c also shows that the tangential component of the solar wind magnetic field was aligned in an opposite direction for the two observations. However, we note that the 10–15 h uncertainty could lead features to be shifted into or out of the observations.

The ability of ACIS to detect soft X‐rays from optically bright, extended targets is hampered by substantial transmission through its optical blocking filters (OBFs) at wavelengths between 0.8 and 0.9 μm. Jupiter at opposition fills some 6000 pixels of an ACIS CCD. In the 1999–2000 observations, each of these pixels received an average charge equivalent to a 140 eV X‐ray. The value has gradually decreased since then—due most probably to contamination buildup on the OBFs. By November 2014, it had fallen to ∼70 eV/pixel, as estimated from observations of Betelgeuse.

To distinguish X‐rays from charged particles passing through the CCDs, an on‐board digital filter scans the charge distribution in each CCD image, seeking local maxima surrounded by charge patterns peculiar to X‐rays. The extra optical signal turns all genuine X‐ray events into nonevents, which are never reported to the ground. The solution, outlined in *Elsner et al.* [2005], has been to (a) take CCD bias frames with Jupiter out of the field of view and (b) increase the digital filter's threshold levels by 140 eV, allowing the software to compensate for the optical signal. During subsequent ground processing, the 5 × 5 block of pixels reported for each event candidate are used to subtract the background charge, including the optical signal.

If the optical contamination were exactly 140 eV/pixel, the energy of an X‐ray could be recovered without any additional systematic error. In practice, Jupiter exhibits strong limb darkening in the near infrared, and most Jovian X‐ray emission comes from the polar regions which are observed close to the limb. Also, the optical point spread function of the Chandra mirrors is strongly diffracted by the intermirror gaps, adding to the limb darkening. The result is that some low‐energy X‐rays, especially those whose charge is split between pixels, are still filtered out. The loss incurred has been estimated by reprocessing a group of eight ACIS observations (a total of 104 ks) of the supernova remnant E0102‐72.3—an extended source similar in angular size to Jupiter, which exhibits a strong low‐energy thermal bremsstrahlung component—adding successive levels of “optical” contamination and measuring the resulting change in low‐energy spectral flux. The correction came to less than 1% for X‐rays above 600 eV, 5 ± 1% at 430 eV, and 10 ± 2% at 220 eV, below which energy the sensitivity of the ACIS CCDs drops off rapidly. To account for this, we applied a correction to the auroral spectra (section 5).

### 2.2 Radio Observations

Alongside Chandra X‐ray observations, a series of multi‐instrument, multiplanet observations were conducted and were initially reported in *Lamy et al.* [2012], including radio observations of Jupiter during the same interval. Using both ground‐based observations, from the Nançay decameter array, and space‐based observations, from WIND, STEREO A and B, Jupiter was found to display intensifications of auroral decametric to hectometric emission close to three successive ICMEs, the second of which is investigated here. These enhancements driven by the solar wind activity were consistent with those evidenced by *Gurnett et al.* [2002] for hectometric emission with Galileo and more recently by *Hess et al.* [2012, 2014] for decametric to hectometric emission from Galileo, Cassini, and Nançay observations.

**Figure 2:**
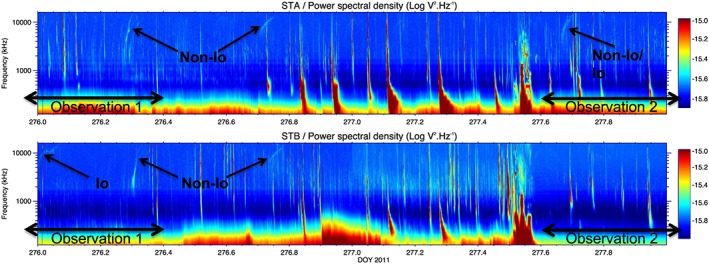
STEREO (top) A and (bottom) B power spectral density plots of the radio emission, shifted for Jupiter‐Earth light travel time (UT 34 min). “Non‐Io” indicates bursts of non‐Io decametric radio emission that suggest the arrival of a forward shock at Jupiter [*Hess et al.*, 2012, 2014]. “Io” indicates Io decametric radio emission associated with activity from Io. The black horizontal arrows indicate the timings of the Chandra X‐ray observations. The first non‐Io decametric burst occurs 0.1 DOY before the end of the first Chandra observation, suggesting that a forward shock arrived at Jupiter during the first X‐ray observation.

The radio observations obtained at the time of the Chandra observations (Figure 2) were shifted to account for light travel time from Jupiter to Earth. Since non‐Io decametric radio emission has been found to be correlated with solar wind pressure [*Hess et al.*, 2012, 2014], investigating this radio emission helps to constrain the arrival time of the ICME‐induced shock.

Non‐Io decametric emission is arc shaped in the time‐frequency plane and the shape of this arc is indicative of the side of the magnetosphere from which it originates. The vertex early or vertex late curvature of these arcs indicates whether the emission source was located westward (Jovian dawn) or eastward (Jovian dusk) of the observer (in the direction of Earth). *Hess et al.* [2012, 2014] showed that forward shocks (where the magnetosphere may be compressed by increased solar wind pressure) are often followed by emission from only one side of the magnetosphere. They showed that reverse shocks (where the solar wind pressure decreases and the magnetosphere may expand) are often followed by emission from both sides of the magnetosphere (i.e., both vertex early and vertex late emission would be observed). At DOY ∼276.3 and 276.7, STEREO A and B data showed two bursts of decametric emission with only vertex early morphology, which suggests the incidence of two solar wind forward shocks at these times. The first of these two bursts coincided with our first X‐ray observation, occurring 2.5 h (0.1 DOY) before the end of the observation (see Figure 2). At ∼276.2 there is also a fainter burst of non‐Io decametric emission.

Two additional radio bursts also featured in the STEREO data: a burst of Io‐D decametric emission at 276.0 and a less intense burst which was only observed by STEREO B (where both spacecraft observed the other bursts) and was difficult to distinguish between Io and non‐Io decametric emission at DOY 277.7. This second indistinguishable burst occurred one Io orbit after the burst on DOY 276.0, which may suggest that Io is the source. If Io is not the source, then it may suggest that a magnetospheric disturbance has been maintained over one Jupiter rotation and that Jupiter's magnetosphere is therefore not completely quiet during the second observation. A corresponding auroral X‐ray enhancement would go undetected for the burst on DOY 276.0 because the auroral footprints had not rotated into view at this time. It would also be very difficult to distinguish the burst on DOY 277.7, since the auroral footprint will have been on the limb of the Jovian disk at this time.

## 3 North Pole Projections

Using the technique applied in *Gladstone et al.* [2002], *Elsner et al.* [2005], and *Branduardi‐Raymont et al.* [2008], time‐tagged Chandra X‐ray events were reregistered into Jupiter's System III (S3) (1965) spherical latitude‐longitude coordinates centered on the rotation poles. Hence, a sky‐projected disk of 1.01 *R*_*J*_ was used for both observations (shown in the supporting information). It should be noted that when reregistering to S3 coordinates, events emitted close to the limb of the Chandra‐facing disk will have larger spatial uncertainties because of the increased obliquity of the planet's surface relative to the observer.

We estimated spatial uncertainties on events based on Chandra's spatial resolution, by perturbing the Jupiter‐centered disk by two pixels in the *x* and *y* directions, then reregistering the events into S3 coordinates.

**Figure 3:**
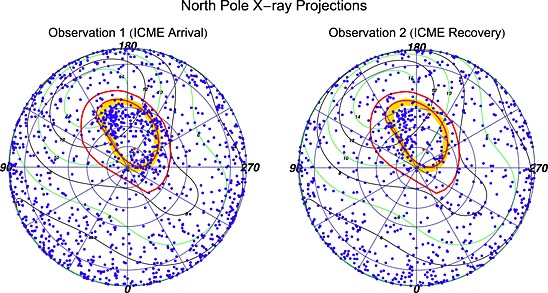
System III (S3) coordinate projections onto Jupiter's geographic north pole (plot center) for the (left) first observation, during which the ICME arrived at Jupiter, and the (right) second observation, 1.2 days later. Lines of constant Jovian S3 longitude radiate outward from the pole, increasing clockwise in increments of 30° from 0° at the bottom of the projection. Concentric dotted circles outward from the pole represent lines of 80°, 70°, 60°, and 30° latitude. The alternate green and black contours indicate VIP4 model magnetic field strength in Gauss. The outer red oval is the *Grodent et al.* [2008] contour of Io's footprint (5.8 *R*_*J*_). The inner red contour is the footprint for the 30 *R*_*J*_ field line from *Vogt et al.* [2011] mapping using the *Grodent et al.* [2008] anomaly model. The thick orange contour is the average location of the UV main oval from two HST observation campaigns in 2007 [*Nichols et al.*, 2009]. The projections show more X‐ray events in the hot spot (160°–180° S3 longitude, 60°–70° latitude) during the first observation than the second. The events appear to spread from the hot spot into the region from 150° to 160°. More clearly identifiable is the bright change in emission in the Auroral Enhancement Quadrant (180°–270° S3 longitude, 55°–90° latitude). The distribution of this emission is not only enhanced in the main oval but also poleward of this and at lower latitudes near Io's magnetic footprint.

**Figure 4:**
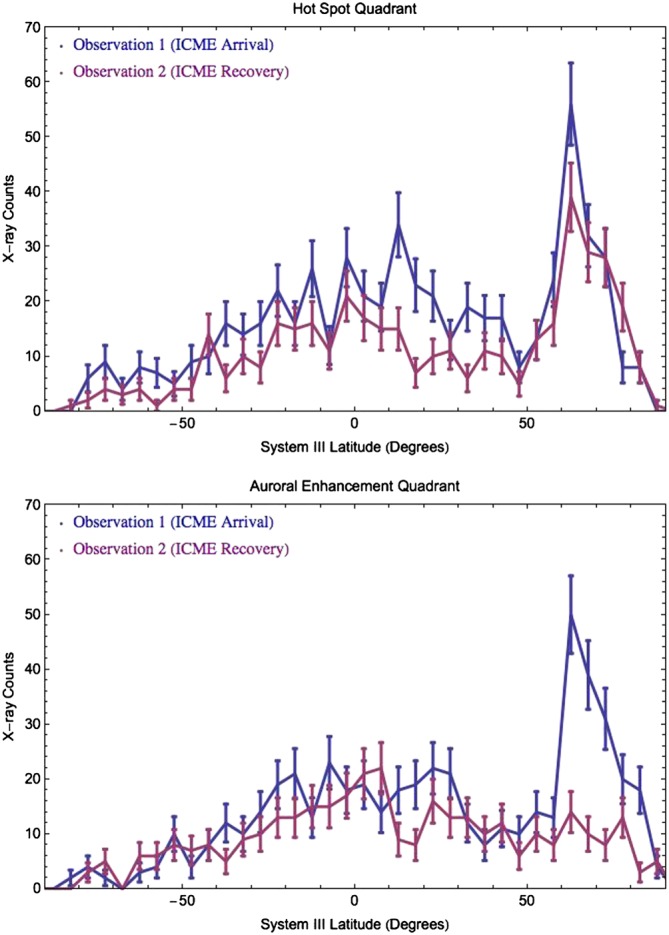
Number of events in 5° latitude bins during the first (blue) and second (red) observations. (top) Hot Spot Quadrant with S3 longitudes 90°–180°. (bottom) Auroral Enhancement Quadrant with longitudes 180°–270°. For the Auroral Enhancement Quadrant, emission above 60° latitude is up to 5 times brighter in the first observation than the second. Error bars are calculated from Poisson statistics. At the time of maximum visibility, each quadrant above 60° latitude had a projected area of ∼3% of the total observable Jovian disk.

To identify the spatial distribution of auroral X‐rays for the two observations, we present projections looking down onto the rotational north pole of Jupiter. Figure 3 shows these projections for both observations. Figure 4 shows counts versus latitude plots to quantify the latitudinal concentrations of X‐rays. During these observations the south pole emission was obscured by the viewing geometry, so we focus on the north pole projections only.

We observe a range of differences in the spatial distribution of X‐rays between the observations (Figures 3 and 4). A surprising difference is a broad bright auroral enhancement in the first observation between 180° and 270° longitude and above 60° latitude. The emission in this area is much dimmer in the second observation. This enhancement is significantly spatially separated from the hot spot (S3 longitude: 160°–180°, latitude 60°–70° [*Gladstone et al.*, 2002; *Elsner et al.*, 2005; *Branduardi‐Raymont et al.*, 2008]), where the brightest X‐ray emission was previously observed. The region above 60° latitude and with longitudes 180°–270° features 201 ± 14 X‐ray counts in the first observation compared to 76 ± 9 counts in the second.

Given the changing solar wind conditions throughout the observations (section 2) and our lack of knowledge concerning the processes governing both the hot spot and the auroral enhancement, we shall analyze the two separately. We refer to the 90°–180° longitude quadrant as the “Hot Spot Quadrant” (HSQ) and to the quadrant between 180° and 270° longitude as the “Auroral Enhancement Quadrant” (AEQ). However, we note that there is brightening across both quadrants and that this may be connected.

We focus first on the HSQ. For both observations, the majority of the auroral emission (above 60° latitude) occurs poleward of the 30 *R*_*J*_ contour (the inner red oval in Figure 3), indicating that the precipitating particles originate farther away from Jupiter than this. The whole region of the HSQ inside the 30 *R*_*J*_ contour contains 113 ± 11 counts in the first observation compared to 78 ± 9 counts in the second. Previously [*Gladstone et al.*, 2002; *Elsner et al.*, 2005], the hot spot was defined as located between 160° and 180° S3 longitude and 60° and 70° latitude, where we find 52 ± 7 counts in the first observation and 37 ± 6 counts in the second observation. We find that the hot spot appears to spread out spatially in the first observation. The outer edge of the hot spot (at longitudes 150°–160° and latitudes 55°–60°) is where the greatest change occurs, with 55 ± 7 X‐ray counts in the first observation compared to 28 ± 5 counts in the second. This changing emission occurs between the 30 *R*_*J*_ contour and the hot spot, in a region which during a 2007 Hubble Space Telescope (HST) observing campaign was where the poleward edge of the UV main oval was observed [*Nichols et al.*, 2009]. The second observation appears to have its events much more concentrated in the previously defined hot spot. UV observations have shown that when solar wind compression regions onset, the UV auroras brighten in the “active region” close to this X‐ray region, near noon and poleward of the main oval [*Grodent et al.*, 2003; *Nichols et al.*, 2007].

**Figure 5:**
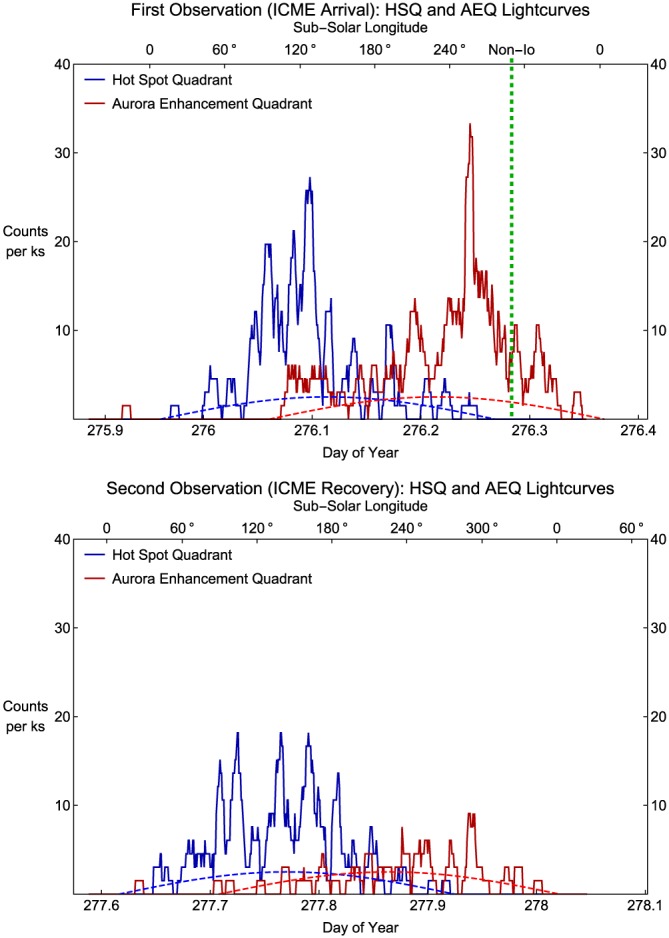
X‐ray aurora lightcurves for the (top) first and (bottom) second observations. Blue line: X‐rays in the Hot Spot Quadrant (S3 longitude: 90–180°). Red line: X‐rays in the Auroral Enhancement Quadrant (S3 longitude: 180–270°). The lightcurves were generated by placing events above 60° latitude in S3 coordinates into 1 min bins. These were then shifted to account for Jupiter‐Earth light travel time of 34 min (UT 34 min). The subsolar longitude at the time of the observations is indicated along the top of each plot. The green vertical dashed line indicates the onset of the brightest burst of non‐Io decametric emission in the STEREO A data. The projected area of each quadrant (as a percentage of the total area of Jupiter) is indicated by the blue (HSQ) and red (AEQ) dashed lines. At the point of maximum visibility each quadrant above 60° latitude takes up a projected area that is ∼3% of the total observable Jovian disk.

For the Auroral Enhancement Quadrant, the first observation displays additional bright features with respect to the second. The difference is most evident in Figure 4, which shows the emission is up to a factor of 5 brighter across all latitude regions from 55° to 85° during the first observation relative to the second. Additionally, Figure 4 shows that in the first observation the levels of emission observed in the AEQ are comparable to those in the same latitude range in the HSQ. Comparing the changes in counts for the HSQ and AEQ could suggest that the HSQ is less sensitive to the ICME than the AEQ. Alternatively, it could suggest that the changes the ICME drives in the X‐ray aurora develop with time or with varying solar wind parameters—as Jupiter rotates, the HSQ is visible first and the AEQ rotates into view slightly later (Figure 5).

One other aspect to note from the HSQ latitude‐count plot (Figure 4) is that there appears to be increased emission from the disk/equatorial region. This suggests the presence of increased solar X‐ray flux, which is fluoresced and elastically scattered in the Jovian atmosphere. The occurrence of a solar flare at a time consistent with the increase is confirmed by inspection of GOES X‐ray lightcurves (see supporting information). Analysis of the polar projections for discrete energy regimes section 6 shows that the flare is not a significant contributing factor for the increased auroral emission, ensuring the validity of the changing auroral activity. We note that this solar flare is a distinct event from the ICME and directly introduces additional solar X‐ray photons to the Jovian disk, while the ICME introduces X‐rays indirectly.

## 4 Auroral X‐Ray Lightcurves

To generate the auroral X‐ray lightcurves, we took those events which occurred above S3 latitudes of 60° in the polar projections (section 3) and placed them into 1 min time bins. We then shifted the lightcurves to account for Jupiter‐Earth light travel time. During the first observation, the X‐ray emission was brighter and more variable with multiple enhancements that contain twice as many counts as similar enhancements in the second observation. To distinguish between variation in emission from the HSQ and the AEQ, we produced separate lightcurves for each quadrant (Figure 5). To help identify any local time dependencies, we also indicate the subsolar longitude (SSL) corresponding to the timing of the observations.

Figure 5 shows that the first half of each observation was dominated by the hot spot. In the first observation, the hot spot became visible shortly before DOY 276.04 and 80° SSL and the counts increased by up to a factor of 6, from ∼4 c/ks to peaks of 19–27 c/ks. For the second observation the hot spot appeared on the face before DOY 277.7 and the counts increased by up to a factor of 4.5, from 4 to 18 c/ks.

The AEQ shows the most striking difference between the lightcurves. The second observation was generally quiet, with ∼3–5 c/ks, with the exception of a single peak containing 9 c/ks at 277.93. In contrast, the first observation contained a prominent single peak of 33 c/ks at DOY 276.24, which lasted 15–25 min and was higher than the peak emission from the hot spot. Prior to the peak, there was a gradual increase from DOY 276.2 to 276.22. After the peak there was an abrupt drop to 17 c/ks and then a gradual decrease for 0.1 DOY afterward, as the region rotated out of view. From the moment the region began to be observable it was emitting 6 c/ks, while in the second observation it emitted only 1–2 c/ks, suggesting that the whole region was brighter throughout the first observation.

The peak of the enhancement occurred 1–1.5 h before the non‐Io decametric radio burst at DOY ∼276.3 (indicated in Figure 5 by the dashed line). We also note that the fainter burst of non‐Io decametric emission at DOY 276.2 coincides well with the preceding peak on the AEQ auroral lightcurve, suggesting a further possible connection between X‐ray emission and non‐Io decametric emission. The previously recognized connections between this non‐Io decametric emission and forward shocks induced by ICMEs [*Hess et al.*, 2012, 2014] suggest that the heightened X‐ray emission is also likely to be directly connected with the ICME.

We also detect periodicity in these lightcurves on the order of tens of minutes for both observations, and this is discussed and analyzed in section 8.

## 5 Auroral Spectra

### 5.1 Spectral Extraction and Modeling

For analysis of the Chandra spectra we divided Jupiter's observed disk emission into three sections: a northern auroral zone, an equatorial region, and a southern auroral zone (see supporting information for regions selected). Given the limited visibility of the southern aurora, only the northern aurora is presented.

Using the CIAO software package (provided by the Chandra X‐ray Center), we followed the standard procedures to extract spectra, which were then analyzed using the XSPEC package [*Arnaud*, 1996]. We applied a correction to the effective area to account for the increased energy thresholds applied within ACIS to circumvent optical light leaks through the OBFs (as discussed in section 2.1). To do this, we weighted energies below 0.7 keV based on fitting for the signal degradation to E0102‐72.3, which provided a best fit curve of 1 − *Y*∗(*x* − 0.7)∗∗2 with *Y* = 0.50 and *x* = the energy of channel.

**Figure 6:**
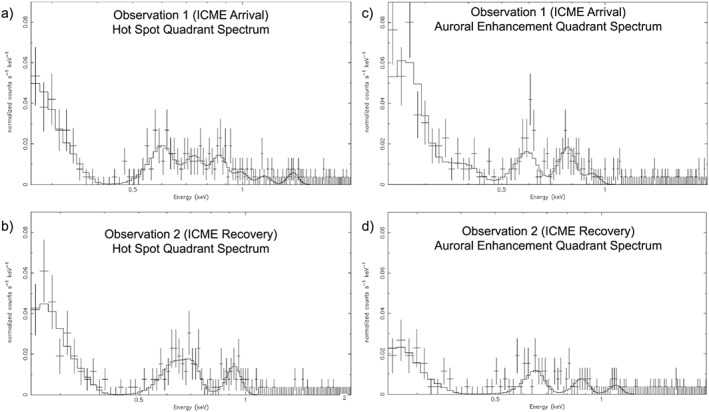
The northern auroral zone spectra for the (a, c) first and (b, d) second observations. The Hot Spot Quadrant spectra are in Figures 6a and 6b, while the Auroral Enhancement Quadrant spectra are in Figures 6c and 6d. The data have been fitted with a combination of lines with half widths fixed at 20 eV.

We again treated the HSQ and AEQ separately. To do this, we separated each observation into two halves based on the time at which the emission from the hot spot dimmed (Figure 5). The spectrum for the first (second) observation HSQ was produced at Jupiter from DOY 275.95 to 276.15 (277.6 to 277.8) UT, while the AEQ events occurred from DOY 276.15 to 276.35 (277.8 to 278) UT. The time intervals were selected to maximize exposure times of the given quadrant, while minimizing contamination from the other. Figures 6a and 6b compare the HSQ spectra, and Figures 6c and 6d compare those from the AEQ, for the two observations.

We fitted the spectra between 240 and 2000 eV, with a combination of lines with half widths fixed at 20 eV. This produced two challenges. First, the low count rates and large error bars produced unrealistically low reduced *χ* ^2^ values of 0.4–0.6 (for 105–111 degrees of freedom). Second, Chandra's spectral resolution and energy cutoff at ∼210 eV lead us to ignore the region from 210 to 250 eV, since the sharp drop in counts in this region inhibited good fitting. Table 1 and Figure 6 show the best fits.

**Table 1:** Best Fit Parameters for the 0.24–2 keV Spectra and Closest Known Ion Rest Frame Lines [*Elsner et al.*, 2005; *Kharchenko et al.*, 2008; *Branduardi‐Raymont et al.*, 2007b]^a^

Best Fit Line (eV)	Flux (photons/cm^2^/s)	Known Ion Rest Frame Energies
*First Observation Hot Spot Quadrant—Reduced χ^2^ ∼ 0.45 (105 Degrees of Freedom)*		
310 ± 10	5 ± 1 × 10^−4^	S VI–X (260–291; 314; 316 eV) or C V (299; 304–308 eV)
595 ± 20	1.5 ± 0.5 × 10^−5^	O VII (561; 568; 574 eV)
730 ± 35	6.5 ± 3 × 10^−6^	O VII (698–713 eV) or O VIII (774 eV)
860 ± 30	4.5 ± 1.5 × 10^−6^	O VIII (836 eV) or Solar Fe XVII (812; 826 eV)
990 ± 60	1.5 ± 1 × 10^−6^	Solar Ne X + Fe XXI (∼1000 eV)
1140 ± 85	9 ± 6 × 10^−7^	Solar Ne X + Fe XXI (∼1000 eV)
1375 ± 60	1 ± 0.5 × 10^−7^	Solar Mg XI (1350 eV)
*Second Observation Hot Spot Quadrant—Reduced χ^2^ ∼ 0.4 (111 Degrees of Freedom)*		
310 ± 10	4.5 ± 1 × 10^−4^	S VI–X (260‐291 eV) or C V (299; 304–308 eV)
610 ± 50	9 ± 5 × 10^−6^	O VII (561; 568;574 eV) or O VIII (654 eV)
700 ± 35	8.5 ± 5.5 × 10^−6^	O VII (698–713 eV)
925 ± 25	4 ± 1 x 10^−6^	Solar Ne X + Fe XXI (∼1000 eV)
*First Observation Aurora Enhancement Quadrant—Reduced χ^2^ ∼ 0.6 (109 Degrees of Freedom)*		
$305_{-100}^{+10}$	3 ± 2 × 10^−4^	S VI–X (260–291 eV) or C V (299; 304–308 eV)
390 ± 60	4.5 ± 3 × 10^−5^	S IX‐ S XIV (336–348; 380 eV) or C V–VI (354–378 eV)
590 ± 15	1.5 ± 0.5 × 10^−5^	O VII (561; 568; 574 eV)
775 ± 20	7 ± 2 × 10^−6^	O VIII (774 eV)
915 ± 65	1.5 ± 2 × 10^−6^	Solar Ne X + Fe XXI (∼1000 eV)
*Second Observation Aurora Enhancement Quadrant—Reduced χ^2^ ∼ 0.55 (111 Degrees of Freedom)*		
310 ± 10	2 ± 1 × 10^−4^	S VI–X (260–291 eV) or C V (299; 304–308 eV)
645 ± 40	7 ± 2.5 × 10^−6^	O VII (665 eV) or O VIII (654 eV; 698–713 eV)
875 ± 60	2 ± 1 × 10^−6^	O VIII (836 eV) or Fe XXI + Ne X (∼1000 eV)
1095 ± 65	1 ± 0.5 × 10^−6^	Solar Ne X + Fe XXI (∼1000 eV)

a Line Half Widths Were Held Constant at 20 eV.

### 5.2 Spectral Analysis

Inspecting the HSQ spectra (Figures 6a and 6b) first, both observations featured a large peak between 250 and 350eV, which could be from sulfur and/or carbon ions.

Between 500 and 900 eV there was a range of oxygen lines. Both observations contained lines near 600 eV and between 700 and 730 eV, which are likely to be from O VII and possibly also O VIII transitions. The first observation showed an additional spectral line at ∼ 860 eV, which could have either been from O VIII transitions or evidence for solar X‐ray scattering from the disk. While the best fit model contained only one line at 730 eV, we were also able to obtain similar reduced *χ* ^2^ values by fitting two lines at ∼700 eV (O VII) and ∼780 eV (O VIII), which may suggest that the additional line at 860 eV was also an O VIII transition.

As mentioned in section 3, a solar X‐ray flare reached Jupiter during the time covered by this spectrum (see supporting information for further details) and may have imprinted solar lines onto the spectrum. The additional emission above 700 eV could have been from Fe XVII, Fe XXI, or Ne X solar photons or a combination of oxygen and solar photons. We also observed a magnesium (Mg XI) line in the spectra near 1350 eV, which would be expected from a solar flare [*Branduardi‐Raymont et al.*, 2007a; *Bhardwaj et al.*, 2005, 2006]. These solar features are absent or much less relevant in the AEQ and throughout the second observation.

For the AEQ, the difference between the spectra of the two observations is clear (Figures 6c and  6d). The first shows a prominent peak between 200 and 300 eV that appears to be 3–4 times higher for the first observation than the second. We were unable to model this accurately because of the low‐energy cutoff and low spectral resolution, meaning that comparing fluxes and differentiating between sulfur and carbon was not possible. Between 300 and 500 eV there are additional transitions of carbon or sulfur which do not appear in the HSQ spectra or the AEQ spectrum for the second observation.

The morphology of the AEQ spectrum between 380 and 700 eV is particularly interesting. The emission between 550 and 600 eV is mostly O VII, and the line appeared to be asymmetric, with a sharp decline after 600 eV, which led the fit to underestimate the flux for this line in Table 1. This region of the spectrum is similar to that of comets LINEAR S4 and McNaught‐Hartley displayed by *Elsner et al.* [2005]. This similarity to cometary solar wind charge exchange spectra could suggest a solar wind origin for some of the precipitating ions.

The 775 eV line appeared to be a good match for the O VIII transition. GOES data (supporting information) shows that the heightened solar X‐ray flux from the first half of the observation was returning to normal at these times, so it is unlikely that solar photons caused the 700–900 eV morphology in this spectrum.

For the AEQ in the second observation, the spectrum is best fitted by a set of low flux sulfur/carbon and oxygen lines. Some of this emission may be contamination from the HSQ, which was still partially visible during these times.

## 6 Connecting Spatial and Spectral Features

Given that Chandra's spectral resolution is insufficient to definitively separate between the spectral lines of carbon and sulfur ions, we now examine the auroral morphology in different energy bands. By combining this with magnetic field mapping, we tried to establish the magnetospheric or solar wind origins for specific ion species. To do this, we binned X‐rays into four broad energy bins for carbon/sulfur, oxygen, solar X‐ray lines, and hard X‐rays. We then plotted the polar projections for each energy range separately. The specific energy ranges were chosen based on (a) the ease with which regions could be differentiated in the spectrum, (b) the relevant spectral lines for different species [*Elsner et al.*, 2005], (c) Chandra's energy resolution limitations, and (d) by considering the solar X‐ray lines from the equatorial region spectrum.

**Figure 7:**
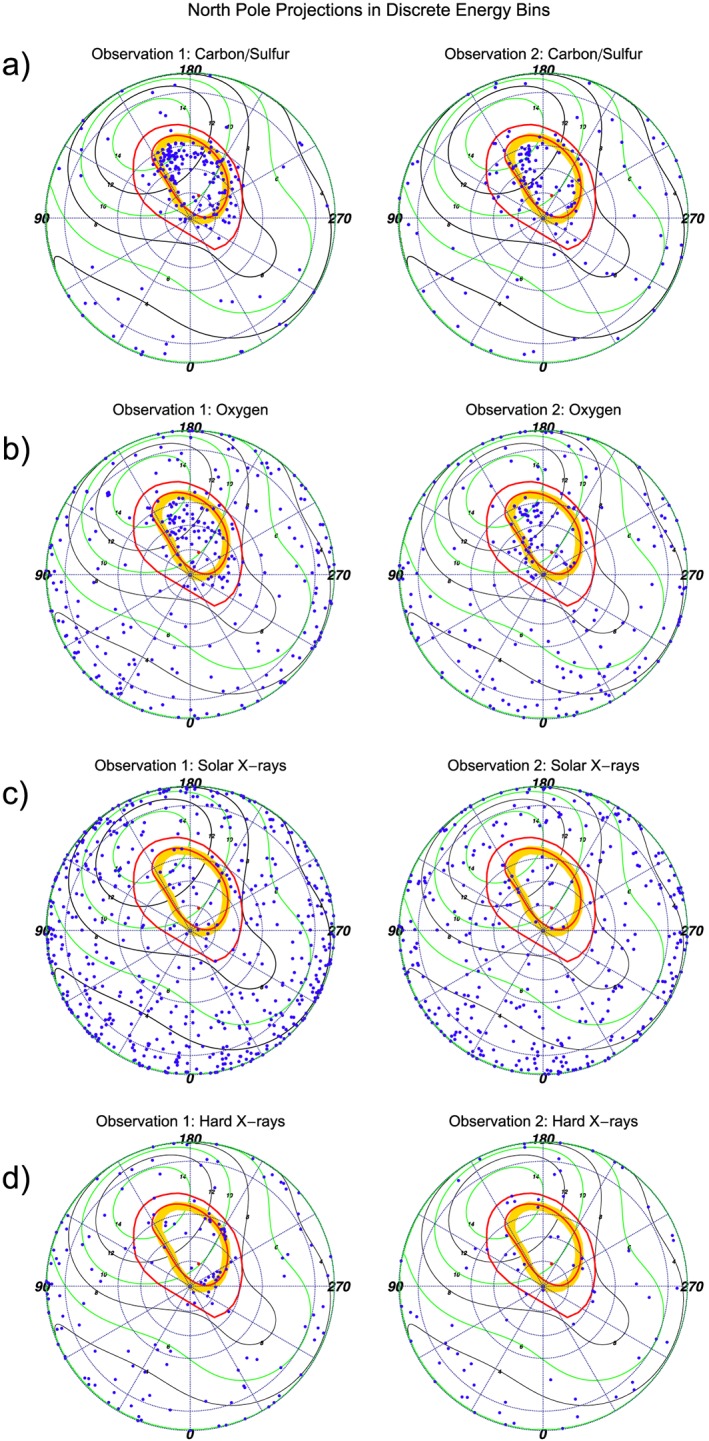
Comparisons of north pole S3 projections for discrete energy ranges for the (left column) first and (right column) second observations. From top to bottom the energy ranges are (a) 200–500 eV (carbon/sulfur ion lines), (b) 500–800 eV (oxygen ion lines), (c) 800–1500 eV (dominated by fluoresced and scattered solar photons), and (d) 1500–5000 eV (hard X‐ray bremsstrahlung radiation from electrons). For further plot details see Figure 3.

We estimated the carbon or sulfur emission from the spectra between ∼200 and 500 eV. We found that photons below 300 eV mapped almost exclusively to the auroral zone, with very little disk component (Figure 7), so we included these photons in our analysis. The oxygen emission was defined by the band ∼500–800 eV from spectral fitting of strong O VII and O VIII lines [*Branduardi‐Raymont et al.*, 2004, 2007b; *Elsner et al.*, 2005]. We considered the ∼800–1500 eV emission to come from fluoresced or scattered solar photons because this energy range contains the peak of the disk spectrum [*Bhardwaj et al.*, 2005, 2006; *Branduardi‐Raymont et al.*, 2007a]. It should be noted that some O VIII lines from completely stripped oxygen [*Elsner et al.*, 2005] also fall in this energy range and may contribute some of the observed auroral emission. Finally, we consider 1500–5000 eV emission to be hard X‐rays from precipitating electrons generating bremsstrahlung radiation [*Branduardi‐Raymont et al.*, 2007b, 2008].

We look first at the polar projections of 200–500 eV carbon/sulfur X‐ray events (Figure 7a) and find that for both observations almost all emission originated in the aurora, with very little equatorial emission. This confirms that the changing emission in this part of the spectra was unrelated to solar flares. We find that carbon/sulfur is the source of the brightening on the edge of the hot spot, between 150° and 160° S3 longitude (introduced in section 3). This emission lies in a region which during the 2007 HST observations [*Nichols et al.*, 2009] featured the poleward edge of the UV main oval.

In the AEQ, for the first observation we find a large number of carbon/sulfur events between the Io footprint (∼5.8 *R*_*J*_) and both the UV main oval and 30 *R*_*J*_ contour. For the AEQ, we also find ion emission poleward of the 30 *R*_*J*_ contour. This is unexpected, since previous observations showed that the majority of ion emission originated in the Hot Spot Quadrant. Emission from carbon/sulfur in the AEQ is largely absent from the second observation.

For the 500–800 eV oxygen emission (Figure 7b), events are also concentrated into the auroral zone. In the first observation, the events occur poleward of the 30 *R*_*J*_ contour and the main oval reference contour in both the HSQ and AEQ, while in the second observation the auroral events are almost solely concentrated into the hot spot. Comparing the oxygen with the carbon/sulfur emission, we find that where there is some carbon/sulfur emission closer to the polar edge of the 30 *R*_*J*_ contour, the oxygen emission generally originates poleward of this carbon‐/sulfur‐dominated emission region and appears to be more diffusely distributed across the entire polar region.

Figure 7c shows the 800–1500 eV emission, dominated by solar photons, distributed across the disk, and not concentrated into the aurora, as expected. The hard X‐rays (Figure 7d) cluster in two regions parallel with the 30 *R*_*J*_ contour in the first observation and are less prevalent in the second.

**Figure 8:**
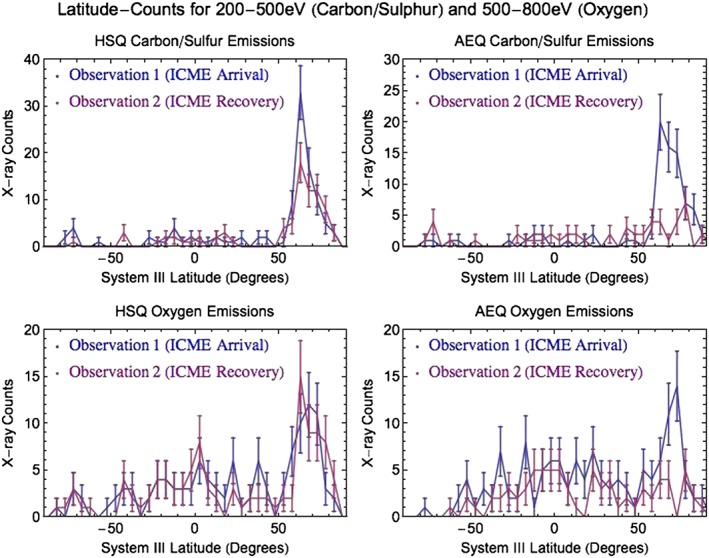
Latitude‐count plots for 5° latitude bins. Comparisons of the (top row) 200–500 eV carbon/sulfur emission or (bottom row) 500–800 eV oxygen emission between the first observation (blue line) and second observation (red line). The (left column) Hot Spot Quadrant and (right column) Auroral Enhancement Quadrant are treated separately. At the time of maximum visibility, each quadrant had a projected area of ∼3% of the total observable Jovian disk.

Figure 8 shows carbon/sulfur and oxygen latitude‐count plots: the change between observations in carbon/sulfur emission is similar in both quadrants, while oxygen emission stays almost constant in the HSQ but changes by a factor of 3 in the AEQ. This differing behavior and mapping for carbon/sulfur emission and oxygen emission may suggest different sources for each.

## 7 Local Time Variation: Noon‐Binned Projections and Magnetosphere Mapping

The configuration of Jupiter's magnetosphere will evolve throughout the observations. As Jupiter rotates, a specific S3 longitude‐latitude auroral position will map to changing magnetospheric local time sources. To identify how this rotation, and the associated change in local time, changes the X‐ray aurora and to identify possible magnetospheric local time origins for features, we mapped the magnetosphere footprint configuration at distinct subsolar longitudes (SSL). The SSL indicates which Jovian S3 longitude is directly facing the Sun at a given time—the location of noon.

To do this, we subdivided each 11 h observation into 50 min time bins. For each time bin, we compared the S3 coordinates of auroral spatial and spectral features with their mapped source regions using the Jovian magnetosphere‐ionosphere model from *Vogt et al.* [2011].

The Vogt model maps contours of constant radial distance from the magnetic equator to the ionosphere by ensuring that magnetic flux at the equator equals magnetic flux in the ionosphere. This enabled us to map ionospheric footprints to their equatorial magnetospheric origins up to 150 *R*_*J*_ from the planet, where the VIP4 model [*Connerney et al.*, 1998] used for previous Jupiter X‐ray observations was limited to 30 *R*_*J*_ [*Gladstone et al.*, 2002; *Elsner et al.*, 2005; *Branduardi‐Raymont et al.*, 2008]. The Vogt model accounts for the bend‐back of Jupiter's field lines, in order to map field lines to their magnetospheric local time origins. For instance, this could inform us that a specific ionospheric footprint maps to an equatorial magnetospheric source 50 *R*_*J*_ from the planet at dawn magnetospheric local time.

Using NASA Jet Propulsion Laboratory Horizons ephemerides data, we chose the start and end times of 50 min X‐ray bins to coincide with 30° increments of SSL. X‐rays emitted at times when the SSL was 15°–45° were compared to the *Vogt et al.* [2011] mapping model at SSL 30° to identify the sources for these X‐rays and so on for each 30° SSL increment.

*Joy et al.* [2002] showed that the magnetopause location of Jupiter is bimodal. During periods of low solar wind dynamic pressure, the nose of the magnetopause standoff is expected to reach ∼92 *R*_*J*_ (an expanded magnetosphere), while for the high dynamic pressure periods, it will be as close as ∼63 *R*_*J*_ (a compressed magnetosphere). *Vogt et al.* [2011] account for these two different possible magnetopause standoff distances by moving the magnetopause location based on the measured distances of *Joy et al.* [2002].

**Figure 9:**
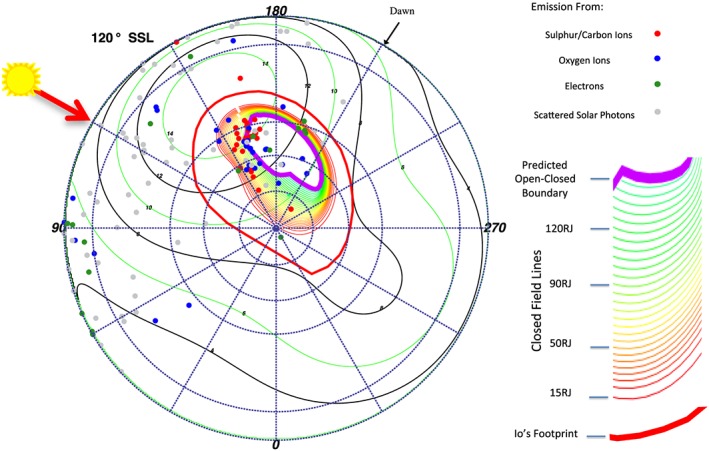
S3 polar projections showing X‐ray emission coinciding with specific subsolar longitudes (SSLs). Each plot shows emission that occurred at times when the SSL was ±15° from the SSL stated (120° in this case). The Sun's direction (noon) lies along the red arrow, with dawn 90° clockwise from this and dusk 90° anticlockwise. A *Vogt et al.* [2011] mapping using a Grodent Anomaly Model [*Grodent et al.*, 2008], assuming an expanded magnetosphere, is plotted onto this polar projection. The plot shows closed field lines increasing in increments of 5 *R*_*J*_ from the 15 *R*_*J*_ contour (red), through 50–80 *R*_*J*_ contours (yellow), to the last closed contour at the nose of an expanded magnetosphere 90 *R*_*J*_ (inner green contour). Green contours map to 95–150 *R*_*J*_. The thick purple contour indicates the predicted open‐closed field line boundary. Regions poleward of this and absent of contours indicate regions mapping to open field lines. Events occurring close to the noon position have uncertainties in their spatial position of ∼5° latitude‐longitude, while those occurring closer to dawn or dusk originate on the limb and have uncertainties of ∼10°–20° latitude‐longitude. Emission is color coded: carbon/sulfur photons (red), oxygen photons (blue), solar X‐rays photons (grey), and hard X‐rays from electrons (green). Carbon/sulfur emission can be found mostly on contours mapping to 50–90 *R*_*J*_ and also clustered in the open field line region. Oxygen emission is mostly on contours of 70–120 *R*_*J*_ and in open field line regions. The hard X‐rays from electrons can be found clustered on the dawn edge of the projection.

**Figure 10:**
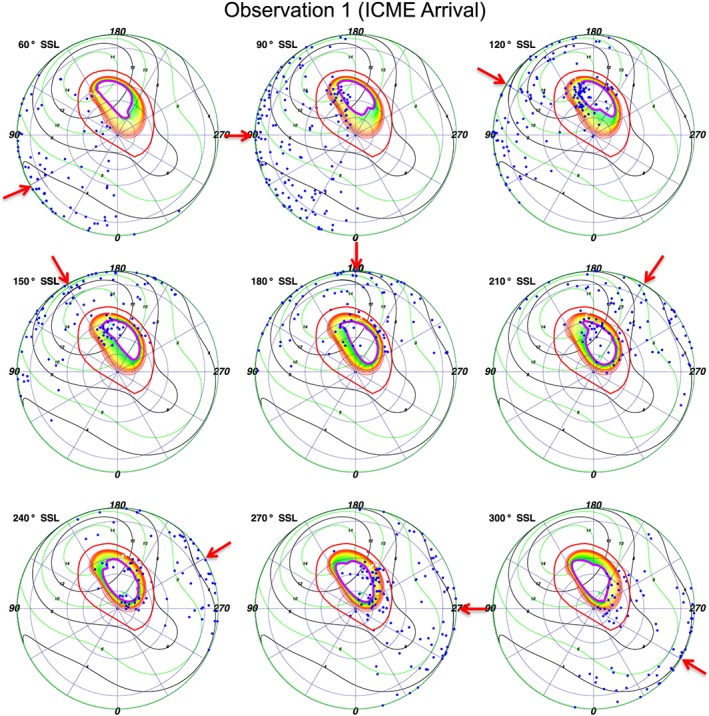
S3 polar projections of the first observation, binned based on subsolar longitude (SSL). *Vogt et al.* [2011] expanded magnetosphere models are plotted onto the polar projections. Throughout the observation, emission appears to exhibit a local time dependence and may follow the open‐closed field line boundary. The time bins at 270° and 300° SSL show the auroral enhancement event. Each dot is an X‐ray photon. For further plot details see Figure 9.

**Figure 11:**
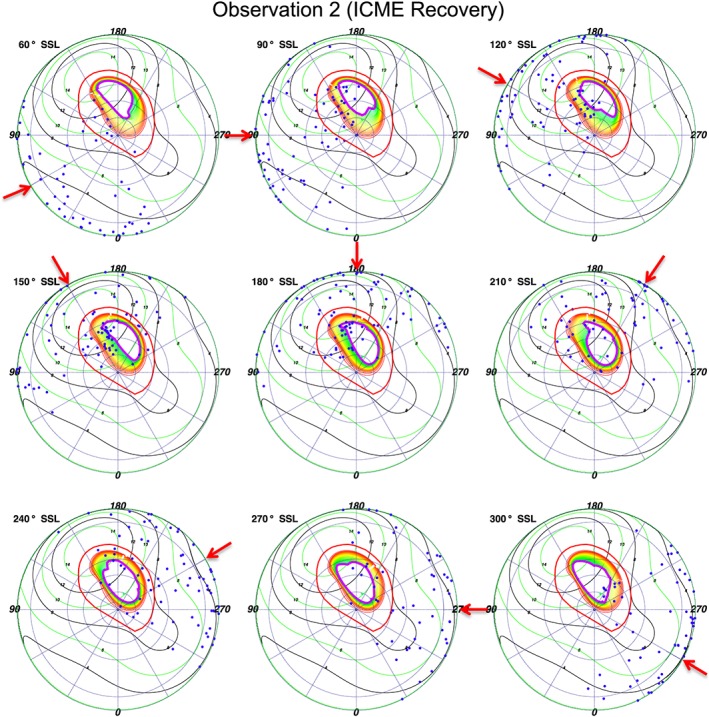
S3 polar projections of the second observation, binned based on subsolar longitude (SSL), with *Vogt et al.* [2011] expanded magnetosphere models. Each dot is an X‐ray photon. For further plot details see Figure 9.

**Figure 12:**
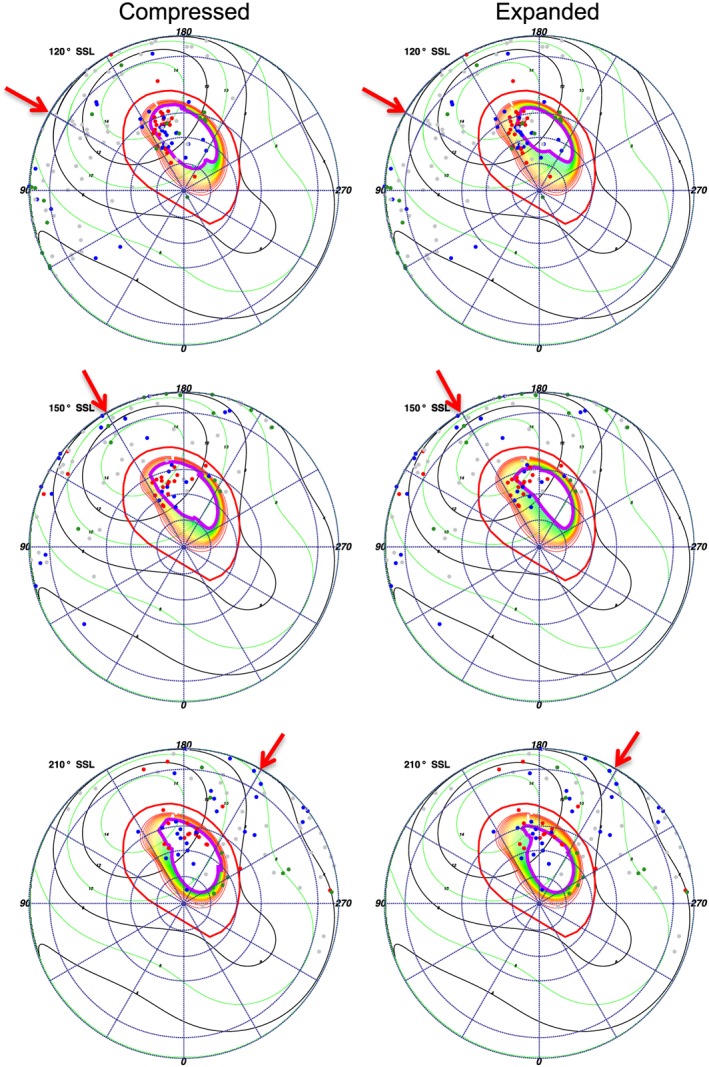
Subsolar longitude (SSL) binned polar projections comparing (left column) compressed and (right column) expanded magnetosphere models for the hot spot during the first observation. Projections for SSL of (top row) 120°, (middle row) 150°, and (bottom row) 210° are shown. The models use *Joy et al.* [2002] measurements of the magnetopause distance. The compressed model uses a noon magnetopause at 63 *R*_*J*_, while the expanded model assumes a noon magnetopause at 92 *R*_*J*_. The field lines increase in increments of 5 *R*_*J*_ from the outer contour of 15 *R*_*J*_ (red), to the final closed inner contour of 65 (yellow—Figure 12 (left column)) or 95 (green—Figure 12 (right column)). For color coding and plot details see Figure 9.

The plotted projections in Figures 9, 10, 11 show the expanded magnetosphere mapping of *Vogt et al.* [2011]. The magnetopause is indicated by a thick purple contour. Jupiter's closed magnetic field lines map to latitudes equatorward of the magnetopause mapping. Toward noon (at the nose of the magnetopause), these closed field lines are shown as contours from 15 *R*_*J*_ (red contour) to 95 *R*_*J*_ (green contour), in increments of 5 *R*_*J*_. For the compressed magnetosphere (Figure 12) closed field line contours at the nose of the magnetosphere extend only as far as 65 *R*_*J*_ (yellow contour). In the Jovian tail we mapped closed field contours up to 150 *R*_*J*_. X‐ray emission that maps to closed contours is likely to be produced by precipitating particles on closed field lines originating in Jupiter's magnetosphere. X‐ray emission that maps poleward of the magnetopause, to the region absent of contours, is from precipitating particles that are more likely to be on open field lines.

Since Jupiter was close to opposition, the SSL and subobserver longitude were only ∼6° separated, so that the noon position on the planet was close to the center of the observed disk. This means that counts originating near the limb of the Chandra‐facing disk are easily identifiable on the time‐binned projections and their larger uncertainties can be accounted for in the context of the magnetic footprint at that moment.

Analyzing the SSL‐binned polar projections with Vogt mapping revealed previously unreported relationships. First, for both the expanded and compressed magnetospheres we find emission that mapped to the open field lines and also emission that mapped to the magnetosphere, suggesting that both could be sources for Jovian auroral X‐rays. For the expanded model (Figures 10 and 11) the majority of the emission originated on the magnetosphere side of the magnetopause, while for the compressed model (Figure 12) the majority of emission originated on open field lines.

This may be particularly noteworthy for the ICME arrival observation. During this observation a compression may be expected to shift the magnetopause boundary from ∼92 *R*_*J*_ to ∼63 *R*_*J*_ [*Joy et al.*, 2002]. It is this region mapping to 60–90 *R*_*J*_, across which the magnetopause would be compressed, which contained the hot spot expansion during the first observation and where we observed increased X‐ray emission. The closeness of the emission to the magnetopause, our spatial uncertainties, and our uncertainty in the choice of expanded or compressed magnetosphere inhibited us from precisely quantifying the relative importance of a solar wind versus a magnetospheric origin. The *Vogt et al.* [2011] models showed, however, that the majority of X‐ray‐producing ions originate beyond 60 *R*_*J*_.

Figures 10 and 11 also show, and particularly for the first observation, that emission clusters along the open‐closed field line boundary and seems to move with SSL, suggesting a local time dependence and relationship with processes in this region. The emission seems to follow the region where field lines would be opening or where closed field lines occur in the afternoon to dusk flank.

### 7.1 Noon‐Binned Hot Spot Projections

**Figure 13:**
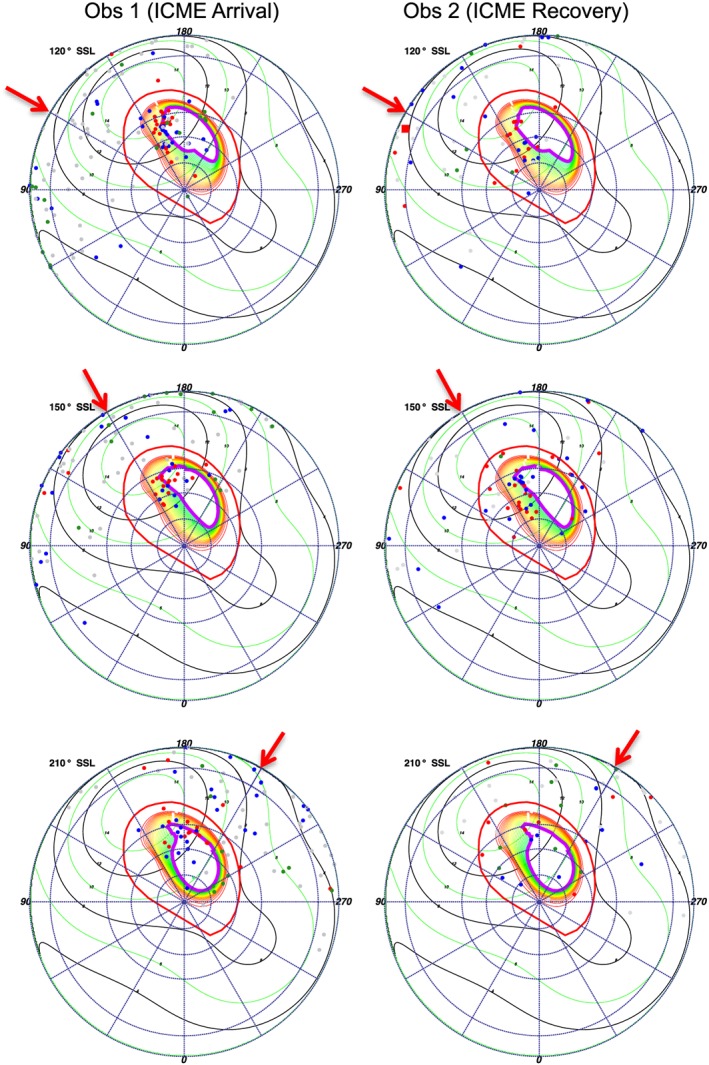
Subsolar longitude (SSL) binned polar projections for the hot spot for the (left column) first and (right column) second observations, using an expanded magnetosphere model for both. For color coding and details see Figure 9.

For our observations, we considered the hot spot to be above 60° latitude and between S3 longitudes 150°–180°. We found for both observations that the hot spot had a strong local time dependence and emitted 78 of 100 X‐rays (first observation) and 51 of 74 X‐rays (second observation) before noon (165° SSL). After this time the hot spot became dimmer, despite the region remaining observable on the Jovian disk for several hours after this. Looking at the development of the magnetic field leading up to 165° SSL (Figure 13), we found that the majority of the hot spot emission originated on the dayside of Jupiter, with magnetospheric local times (MLTs) between 10:30 and 18:00. Later in the observation, when the field lines that mapped to MLTs after 18:00 were still observable in the hot spot, we found significantly less emission from the region.

Having found that the hot spot emission occurred predominantly in the projections 90°–150° SSL (Figures 10 and 11) (prior to mapping to MLTs of 18:00), we analyzed these more closely. For the 90° SSL projection, the hot spot was close to the limb of the disk, so there was a large uncertainty of 10°–20° in the X‐ray coordinates. Based on this, we focused our attention on projections of 120° and 150° SSL (Figures 12 and 13), where the uncertainty was closer to 5° latitude‐longitude.

Considering the first observation 120° SSL projection (Figures 12 and 13), in the region of 150°–170° longitude and 55°–80° latitude, carbon or sulfur (red) emission and oxygen (blue) emission occurred along the field line contours. For the compressed magnetosphere, both carbon/sulfur and oxygen ions originated along the open edge of the open‐closed field line boundary, while for the expanded magnetosphere the carbon/sulfur ions originated on closed field lines. Accounting for spatial uncertainties, the carbon/sulfur events originated between 50 and 90 *R*_*J*_ (yellow‐green contours) and on open field lines, while the oxygen ions originated poleward of this between 70 and 120 *R*_*J*_ (green contours) and also on open field lines. The emission was weaker in the second observation for this SSL projection (Figure 13).

For the 150° SSL projection, both observations (Figure 13) contained clustering of X‐rays between 160 and 170° S3 longitude and 60 and 70° latitude from the afternoon‐dusk flank of the magnetosphere [*Vogt et al.*, 2011]. Given that the time binning is broad (50 min) across 30° SSL, it is uncertain whether these field lines were open or closed for most of this X‐ray emission. Considering uncertainties in the spatial location, this region would map either to the solar wind or closed field lines between 90 and 150 *R*_*J*_. The similar source in both 120° and 150° SSL may suggest that the processes are persistent.

Finally, inspecting the 210° SSL projection (Figure 13), we found that the hot spot contained very little emission, despite remaining on the observable disk. The emission appeared to have followed those field lines that mapped to MLT regions from 12:00 to 18:00 as Jupiter rotated, and we found emission in both the outer magnetosphere and on open field lines in this area.

To reflect our spatial uncertainties, the timing spread of events and their broad spatial distribution in each region, we found a broad range of MLT sources for the emission. For the 120° and 150° SSL projection, most ion emission originated from magnetosphere locations with local times between 10:30 and 18:00. For the 210° SSL projection, events mapped to MLTs of 8:30–19:00 (Figure 13). However, we note that none of these MLTs account for ion travel time from regions near the magnetopause to Jupiter's pole. During this time, the magnetosphere will rotate and so the origins for the particles may be at earlier MLTs than we have suggested. Without knowing the location of the energization region for the ions, it is difficult to quantify this time lag.

### 7.2 Noon‐Binned Auroral Enhancement Projections

**Figure 14:**
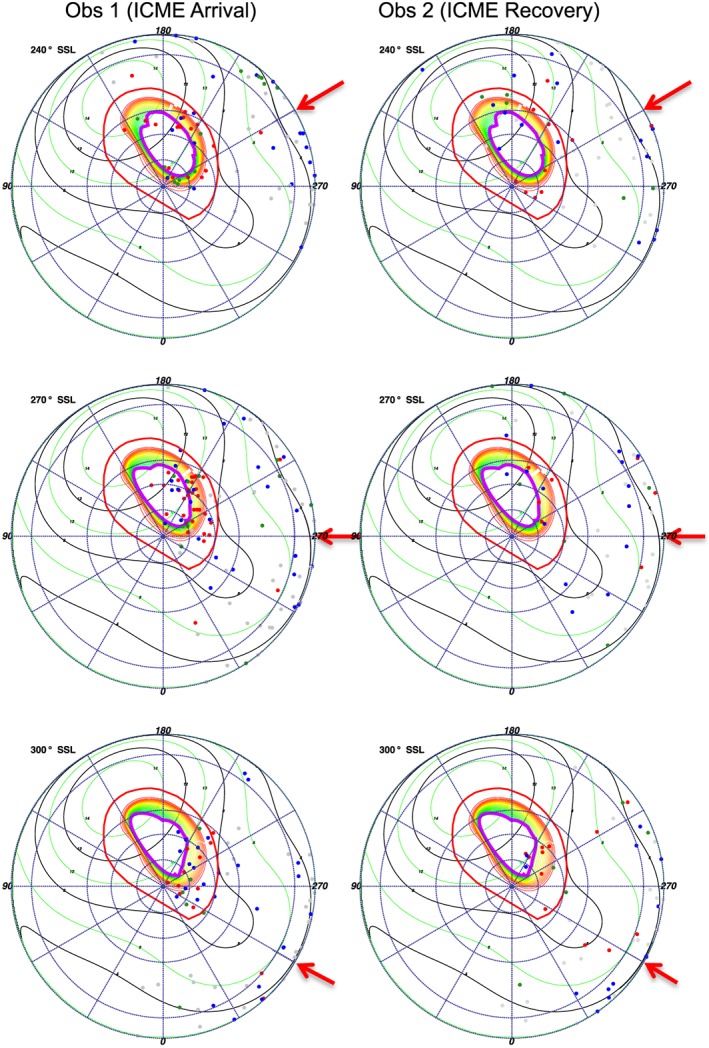
Subsolar longitude (SSL) binned polar projections for the auroral enhancement for the (left column) first and (right column) second observations, using the expanded magnetosphere model for both. The auroral enhancement occurs in the 270° SSL plot. For color coding and plot details see Figure 9.

To identify the source(s) and development of the auroral enhancement, we focus on the 240°, 270°, and 300° SSL projections (Figure 14). Unfortunately, the auroral region had just begun to rotate out of view at this time, so a lot of the brightening occurred close to the limb of the disk, meaning that there were uncertainties of 10°–20° on the S3 coordinates of many X‐rays.

The 270° SSL projection, when the auroral enhancement occurred, contained a broad spread of emission from closed lines in the outer magnetosphere and field lines that were open to the solar wind. This showed both oxygen and carbon/sulfur emissions from the open field line region. The emissions broadly mapped across the dayside of the planet between 06:00 and 16:00 MLT.

The 300° SSL projection had almost all the emission close to the limb, making it challenging to determine the location of the events because of the S3 uncertainties. Carbon/sulfur and oxygen emissions appeared to originate from the magnetosphere, from lower latitude regions than the 15 *R*_*J*_ footprint and from the open regions.

While we cautiously note that the counts were much lower for the hard X‐ray emission from electrons (green), the hard X‐rays appeared to cluster on the dawnside of the disk. This can be seen on the polar projections for SSLs 120°, 210°, and 240° (Figures 13 and 14). These regions mapped to MLTs 02:00–06:30 h. This is on the opposite side of the magnetosphere to the origin for the precipitating ions but is consistent with the vertex early dawn origin for the non‐Io decametric emission that is observed coincident with the first observation and which is also produced by electrons.

## 8 Timing Variation and Periodicity

Following the lead of *Gladstone et al.* [2002] and *Elsner et al.* [2005], we searched the observations for periodicities by selecting a circle (radius: 6.5°, center: 67° latitude, 170° longitude—see supporting information for further details) in S3 coordinates centered on the hot spot and then Fourier transformed the lightcurve from this region to generate power spectral density (PSD) plots. We found that the area used by *Gladstone et al.* [2002] and *Elsner et al.* [2005] showed periodicity at two significant timescales during our first observation, at 12 and 26 min. Their significance increased by expanding the circle to a radius of 8°, centered on 65° latitude and 163° S3 longitude. This larger region included more of the broad spatial spread of hot spot emission in the first observation, showing that the period was also present in the emission between the hot spot and 50 *R*_*J*_ contour. For the second observation, we found that the most statistically significant period occurred using the same S3 circle as *Gladstone et al.* [2002] and *Elsner et al.* [2005].

**Figure 15:**
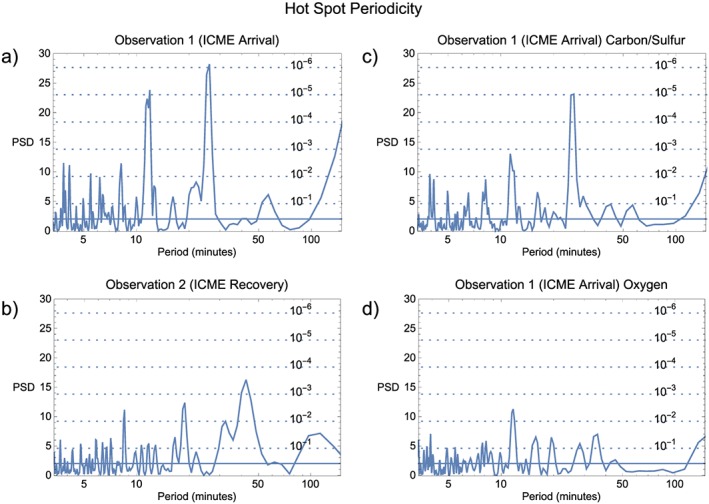
Power spectral density (PSD) plots showing periodicity in the hot spot: (a) Observation 1 (ICME arrival), (b) observation 2 (ICME recovery), (c) observation 1 sulfur/carbon (200–500 eV) photons, and (d) observation 1 oxygen (500–800 eV) photons. During the first observation two periods were detected at 12 and 26 min. The 26 min peak was more significant than the 45 min period reported by *Gladstone et al.* [2002]. The second observation contains a less distinctive periodicity, with the most prominent period at 42 min. The hot spot region was found to be much broader during the first observation, so a different region was used for each PSD to maximize the significance of the periods and to utilize as much emissions from the expanded hot spot as possible (see text for details). Carbon/sulfur emissions are dominated by the 26 min period and also feature a less significant 12 min period. The oxygen emissions feature no 26 min but do feature the less significant 12 min period. When the two populations are combined, the 12 min period becomes much more significant. The dotted horizontal lines show single‐frequency probabilities of chance occurrence (PCO) for the detected periods [*Leahy et al.*, 1983]. The lowest statistical significance and therefore highest PCO of 10^−1^ is at the bottom of the plot, and the highest statistical significance and therefore lowest PCO of 10^−6^ is toward the top of the plot.

To estimate the single‐frequency probability of chance occurrence (PCO) for the detected periods, we used the statistical methods of *Leahy et al.* [1983]. The results are shown as dotted horizontal lines in Figures 15a–15d. The lowest statistical significance and therefore highest PCO of 10^−1^ is at the bottom of the plot, and the highest statistical significance and therefore lowest PCO of 10^−6^ is toward the top of the plot.

For the first observation, we found two strong periods (Figure 15a). The most prominent period occurred with a period of 26 min and a PCO of less than 10^−6^. This is shorter and more significant than the *Gladstone et al.* [2002] period (∼45 min, 4 × 10^−6^). The second period had a timescale of 12 min and a PCO of 10^−5^. We tested a range of locations and sizes of regions encompassing the hot spot and found that these two periods dominated, although which of the peaks was most dominant did swap. The 26 min peak was more dominant on the edge of the hot spot, where the carbon/sulfur particles were more concentrated than oxygen. The 12 min period was more dominant above 70° latitude where the carbon/sulfur and oxygen are more evenly distributed.

Periodicities in the second observation were weaker than in the first (Figure 15b). The most prominent period was at 42 min, with a PCO of 5 × 10^−4^, not as significant as the period in the first observation or that reported by *Gladstone et al.* [2002]. There was also indication of a shorter period of 19 min, but this was even lower in significance.

To determine whether one period was associated with one particle population, we used the same 8° radius region centered on 65° latitude and 163° S3 longitude and generated PSDs for discrete energy ranges. Figure 15c shows a prominent 26 min period at high significance for the carbon/sulfur ions, with a PCO of 10^−5^. It also shows a much weaker 12 min period with a PCO of 2 × 10^−3^. Conversely, the oxygen emission (Figure 15d) exhibited no 26 min period, and the strongest period was at 12 min with a PCO of 5 × 10^−3^. This suggests that one dominant sulfur/carbon population produced the 26 min period, while a second combined population of sulfur/carbon and oxygen generated the 12 min period. For the second observation, the number of X‐ray events was too low to provide reliable results when separating the carbon/sulfur and oxygen populations. The paucity of hard X‐rays from precipitating electrons also made it difficult to identify a significant period for them, although there is a suggestion of some 5–10 min periodicity for the first observation (see supporting information). We also tested regions across the rest of the auroral zone and disk and found no other significant periods (see supporting information).

The two periods in the first observation could have been due to harmonics, although in this case it is challenging to explain how the period is divided between the two separate particle populations in this manner. This division by energy also suggests that they are unlikely to be from instrumental influence.

## 9 Summary of Results

We summarize results separately for the Hot Spot Quadrant (S3 longitude: 90–180°) and the Auroral Enhancement Quadrant (S3 longitude: 180–270°), since solar wind conditions may have been different for each (see Figure 1) and the spatial, spectral and temporal features differ.

### 9.1 Hot Spot Quadrant

1. *Spatial Emission*. The change in emission in the hot spot is not as significant as in the AEQ (Figure 4). This increased emission is concentrated between the previously reported hot spot location [*Gladstone et al.*, 2002; *Elsner et al.*, 2005] and the 50 *R*_*J*_ footprint. This gives the appearance of the hot spot having expanded for the first observation.
2. *Spectra*. Both observations feature prominent 200–400 eV carbon/sulfur peaks and a prominent peak in the O VII spectral region between 550 and 620 eV. The first observation features either increased O VIII emission or increased solar photon emission.
3. *Energy‐Binned Polar Projections (Figure* 7). The 200–500 eV (carbon/sulfur) emission is mostly responsible for the increased emission between the normal hot spot location and the 50 *R*_*J*_ footprint in the first observation. Generally, 500–800 eV (oxygen) emission occurs poleward of this concentrated carbon/sulfur emission. We also find that the carbon/sulfur emission does not behave like the oxygen emission, with the carbon/sulfur emission brightness more enhanced than the oxygen emission for this expanded hot spot.
4. *SSL projections with Vogt et al. [* 2011 *] model mapping*. 78% (first observation) and 69% (second observation) of hot spot emission occurs before noon in the region. This timing coincides with the region mapping to magnetospheric local times between 10:30 and 18:00 h. Most of the carbon/sulfur emission originates in the outer magnetosphere between 50 and 90 *R*_*J*_ and on open field lines, while the oxygen emission originates farther from Jupiter (70–120 *R*_*J*_) or on open field lines (with identification of an open or closed origin depending on uncertainties in spatial resolution and choice of compressed/expanded magnetosphere mapping). The expansion of the hot spot occurs on field lines mapping to the region where the magnetopause has been found to move during compression from 92 *R*_*J*_ to 63 *R*_*J*_ [*Joy et al.*, 2002]. The *Vogt et al.* [2011] model mapping showed that the majority of X‐ray‐producing ions originate beyond 60 *R*_*J*_.
5. *PSDs*. The first observation features two significant periods at 12 and 26 min—shorter timescales than previously reported [*Gladstone et al.*, 2002]. The second observation shows a less significant period of 42 min, closer to the 45 min timescale of *Gladstone et al.* [2002]. The 26 min period is strong in carbon/sulfur emission in the hot spot, but not in oxygen emission. The 12 min period is present for both carbon/sulfur and oxygen, but with much lower significance for each. When the two populations are combined the period becomes significant.

### 9.2 Auroral Enhancement Quadrant

1. *Lightcurves*. An auroral enhancement occurs during the first observation, the peak of which is ∼8 times brighter than for emission in the region during the second observation. This occurs 1‐1.5 h before a non‐Io decametric radio burst, a previously recognized signature of ICME‐induced forward shocks [*Hess et al.*, 2012, 2014; *Lamy et al.*, 2012].
2. *Spectra*. The spectra from the first and second observations are different: there is an enhanced 200–400 eV carbon/sulfur double peak and a prominent peak in the O VII spectral region between 550 and 620 eV during the first observation. These peaks are much less prominent in the second observation. Between 380 and 700 eV the spectrum appears similar to cometary spectra from solar wind charge exchange [*Elsner et al.*, 2005].
3. *Energy‐Binned Polar Projections*. Both the 200–500 eV (carbon/sulfur) and 500–800 eV (oxygen) emissions are increased by a factor of at least 4 for both energy ranges in the first observation relative to the second. This is different to the hot spot emission, where carbon/sulfur is preferentially enhanced.
4. *SSL projections with Vogt et al. [* 2011 *] model mapping*. The enhancements broadly map across the dayside of the planet between 06:00 and 16:00 MLT parallel with the open‐closed boundary. The emission maps to open field lines and closed field lines in the outer magnetosphere and also to low‐latitude regions between Io's footprint and the 15 *R*_*J*_ contour.
5. *Hard X‐rays*. The 1500–5000 eV (electron bremsstrahlung) emission is observed in clusters in the main oval region. It coincides with dawn on the surface and originates at MLT 02:00–06:30 h. This is on the opposite side of the magnetosphere to the source of the X‐ray charge exchanging ions.
6. *PSDs*. No significant periodicity was detected from the AEQ ion emission.

## 10 Discussion

In the discussion, we attempt to address the following questions: What are the source regions for Jupiter's X‐ray aurora? What processes in these regions produce X‐rays and how might these relate to the ICME?

The spectral, spatial, and temporal differences between the hot spot and the auroral enhancement emission lead us to treat the two features separately. Our analysis of the periodicity, spectral and spatial origins of the emission, suggests that both the hot spot and auroral enhancement each have multiple X‐ray sources regions.

Throughout the first observation, the SSL‐binned projections with *Vogt et al.* [2011] mapping show clustering of ion precipitation in the open field line region (Figure 10). This appears to indicate that there is at least some level of precipitation of ions from both the open and closed field lines throughout the first observation. This is less clear for the second observation, where there appears to be lower levels of open field line emission and more is instead concentrated on closed field lines. The *Vogt et al.* [2011] models showed that the majority of X‐ray‐producing ions originate beyond 60 *R*_*J*_. If we assume a compressed magnetosphere (with a standoff distance at 63 *R*_*J*_ [*Joy et al.*, 2002]), the open field lines therefore contribute a large proportion of the emission, while for an expanded magnetosphere (with a standoff distance at 92 *R*_*J*_ [*Joy et al.*, 2002]), closed field lines are the dominant source.

### 10.1 The X‐Ray Hot Spot

#### 10.1.1 Where Is the Hot Spot Source?

While the auroral enhancement emission appeared to originate from several regions that map to different magnetospheric locations, the hot spot remained confined to a more limited region fixed in the planet's rotating frame. This spatial confinement permits more precise identification of possible sources for the precipitating ions that produce X‐ray emission in this region.

The 200–500 eV sulfur and/or carbon emission features an additional component from lower latitudes than the 500–800 eV oxygen emission. If we assume an expanded magnetosphere, we find that most of the 200–500 eV emission maps to a region between the outer magnetosphere and the magnetopause, originating between 50 and 90 *R*_*J*_ (Figure 12). This model suggests that most 200–500 eV emission is from precipitation of high charge‐state sulfur ions in the outer magnetosphere, as proposed by *Cravens et al.* [2003]. It also suggests that there may be some slight precipitation from open field lines and therefore possibly from carbon ions in the solar wind but that this is a smaller proportion of the emission. However, in the case of a compressed magnetosphere, the emission originates is more evenly distributed between carbon ions in the solar wind and from sulfur ions from the outer edge of the magnetosphere (for a compressed magnetosphere this is 50–63 *R*_*J*_ at the standoff point).

The observed strong 26 min periodicity for these 200–500 eV X‐rays may support a sulfur source, since if the period originated in the solar wind, we would expect to also observe oxygen exhibiting it (as the most abundant heavy ion in the solar wind). The absence of oxygen emission from the 26 min period and spatial separation between these two species suggests that the lower latitude feature is from a dominant sulfur population, which does not include oxygen of a sufficiently high charge state. The 12 min period increases in significance when oxygen is combined with carbon/sulfur, suggesting that there is a second population consisting of a mixture of both oxygen and carbon/sulfur. Alongside the periodicity, the spatial mapping suggests a different origin for each population: one solely sulfur population with 26 min periodicity from 50 to 70 *R*_*J*_ and the other an oxygen + carbon/sulfur population from closer to the magnetopause and possibly from open field lines. Comparison of the two observations would seem to suggest that the lower latitude sulfur‐dominated population is more sensitive to changes in the solar wind conditions, since it is much more prevalent in the first observation.

Io injects both oxygen and sulfur into the Jovian magnetosphere, so if both X‐ray‐producing populations originate in the outer magnetosphere, there needs to be an explanation for why the 50–70 *R*_*J*_ region is dominated by sulfur emission and features less oxygen emission. Oxygen ions that produce X‐rays have a higher ionization energy than sulfur ions. For instance, O^6+^ requires 739 eV to become ionized [*Drake*, 1988], while S^6+^‐S^9+^ only requires 281–447 eV [*Biémont et al.*, 1999]. This means that it is possible to have a magnetospheric region where there is sufficient energy for charge stripping and X‐ray production from sulfur, but not from oxygen. More energy would be expected to be injected closer to the magnetopause either through pulsed dayside reconnection, where the field lines closer to the magnetopause would be more perturbed [*Bunce et al.*, 2004], or through field‐aligned potentials [*Cravens et al.*, 2003], which would be expected to increase with radial distance from Jupiter. It is therefore possible that either of these mechanisms could create a higher‐energy region closer to the magnetopause and a lower‐energy region deeper into the magnetosphere. It is also possible that quenching and opacity effects, as suggested by *Kharchenko et al.* [2008] and *Ozak et al.* [2010], may need to be considered to explain the spatial and periodic differences between the two populations.

**Figure 16:**
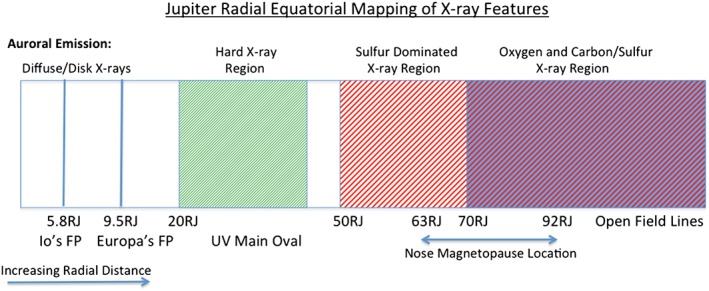
Summary of X‐ray source mapping (not to scale) accounting for uncertainties in photon spatial mapping. The *x* axis indicates the equatorial radial distance from Jupiter that the source regions map to. The different X‐ray regions are indicated by the striped blocks: the hard X‐ray region (green), the region dominated by high charge‐state sulfur region (red), and the mixed high charge‐state carbon/sulfur and oxygen regions (red and blue).

Figure 16 summarizes the equatorial mapping of the sources for different precipitating particles generating the observed X‐rays. Findings from recent work by T. Kimura et al. (Dynamics and source location of Jupiter's high energy X‐ray aurora investigated by Chandra, XMM‐Newton and Hisaki satellite, manuscript in preparation) also identify similar sources for X‐rays and identify both closed field lines in the outer magnetosphere and open field lines beyond the magnetopause as possible X‐ray sources.

The presence of both magnetospheric and cusp precipitation is not precluded by the findings of *Cravens et al.* [2003], *Bunce et al.* [2004], or *Kharchenko et al.* [2006, 2008], but cusp precipitation would only be the dominant source of emission during auroral UV flare‐like conditions or heightened solar wind conditions. The mSWiM propagation and radio emission show solar wind densities increased at Jupiter during the first observation, suggesting that these heightened solar wind conditions may have been present. Cusp precipitation would include precipitation from protons, which are highly abundant in the solar wind and would be expected to generate bright polar UV flares [*Cravens et al.*, 2003]. Without coincident UV observations at the time of the X‐ray observations reported here, it is difficult to identify levels of proton precipitation and therefore to further separate a solar wind or magnetosphere source for the higher‐latitude mixed population of high charge‐state oxygen and carbon/sulfur.

The precise magnetospheric origins of each particle depends on not only the spatial uncertainties but also the internal field model used to initialize the *Vogt et al.* [2011] mapping. *Vogt et al.* [2015] analyzes the differences in each model (VIP4 [*Connerney et al.*, 1998], Grodent Anomaly Model [*Grodent et al.*, 2008] and VIP Anomaly Longitude [*Hess et al.*, 2011]) and highlights the differences between each. From a simple X‐ray hot spot comparison, we found that the Grodent Anomaly Model we used in this work normally mapped X‐rays closer to Jupiter. VIPAL and VIP4 often mapped emission beyond the magnetopause. When the Grodent Anomaly Model did map X‐rays more distantly than VIP4, then there was often less than 10 *R*_*J*_ separation and local times were often 0.5–3 h later than VIP4 or VIPAL.

#### 10.1.2 What Process Drives the Hot Spot X‐Ray Emission?

We find that in both observations the ions that precipitate to produce the hot spot originate from locations between 10:30 and 18:30 on the dayside magnetosphere. Particularly, we find that emission occurs alongside locations where recently opened field lines may occur or on closed field lines in the afternoon flank (but still close to the magnetopause and on the dayside of the planet). *Bonfond et al.* [2011] map quasiperiodic auroral flares in the far UV to the same region in Jupiter's magnetosphere at local times between 10:00 and 18:00 and note the similarity between these flares and flux transfer events observed by Pioneer and Voyager probes. They suggest possible connections between these UV and X‐ray features and the Jovian cusp.

Combined with the dayside origin, the periodicities observed may also be a clue to the mechanisms driving the emission. Using Ulysses, *Marhavilas et al.* [2001] found dual periods of 15–20 min and 40 min in energetic particles upstream from the Jovian bow shock. This may indicate a solar wind connection for emission. Ulysses also detected 20 min and 40 min periodicities in the dusk magnetosphere [*Anagnostopoulos et al.*, 1998, 2001; *Karanikola et al.*, 2004]. Alternatively, the 12 min period falls within the 10–20 min timescale of Jovian global ultra‐low‐frequency oscillations [*Khurana and Kivelson*, 1989]. High‐energy ions have also been previously observed to have periods within this range [*Wilson and Dougherty*, 2000]. At Earth, ultra‐low‐frequency waves are often associated with dayside reconnection [*Prikryl et al.*, 1998] or with either compression from shock events or Kelvin‐Helmholtz instabilities [*Dungey and Loughhead*, 1954; *Chandrasekhar*, 1961; *Kivelson and Russell*, 1995]. It seems possible that one or more of these mechanisms may contribute to the detected hot spot periods in our observations.

*Bunce et al.* [2004] found that pulsed dayside reconnection perturbing outer magnetosphere field lines would generate arc‐like emission and an ∼30–50 min period, not dissimilar to the 26 min period we observe. They also suggest that this is more likely to occur during high solar wind pressure, such as during our first observation. At this time, in support of a reconnection origin, emission appears to cluster close to regions where reconnection could occur (Figure 10). *Desroche et al.* [2012] found that reconnection was possible in the afternoon to dusk region based on plasma flow shear speeds, *β* = 10 and *β* = 1, which may suggest that the local time dependence of hot spot emission could be connected with this process.

If the 26 min periodicity were to be related to bounce times on field‐aligned potentials instead, then it remains challenging to explain the shared 12 min oxygen and carbon/sulfur periods in this way. This is because the different masses of oxygen and sulfur/carbon would produce different bounce times for ions that originated in the same region. Their shared period may therefore favor a non–bounce time‐related mechanism for the 12 min period in the first observation. We note that this 12 min period is of the same order of magnitude as the Alfvèn wave transit times calculated by *Bunce et al.* [2004]. If the periodicity does relate to the Alfvèn transit time, then the shift in period from 12 or 26 min to 42 min may make sense in the context of a shift in magnetopause distance because of solar wind‐induced compression/expansion of the magnetosphere.

For the second observation, when the solar wind was returning to pre‐ICME conditions, emission still originates from the dayside of the planet but more prominently from locations in the magnetosphere closer to 15:00–18:00 MLT, along recently closed field lines (Figure 13). Kimura et al. (manuscript in preparation) suggest that flow shear effects such as Kelvin‐Helmholtz instabilities (KHIs), also found at the magnetopauses of Saturn [*Masters et al.*, 2010; *Wilson et al.*, 2012] and Earth [*Hasegawa et al.*, 2004], may be an important factor, and thus an explanation for the periodicity in the Jovian X‐ray emission. KHIs are expected to develop on both the dawn and dusk flanks of the planet and are expected to become more substantial moving down the flanks, where the velocity shears are larger, as the magnetosphere and solar wind become progressively more rolled‐up [*Miura*, 1984; *Nykyri et al.*, 2006]. These structures could either inject solar wind particles directly into the magnetosphere, through small‐scale reconnection events [*Fairfield et al.*, 2000; *Nykyri and Otto*, 2001], or could facilitate the transport of momentum across the magnetopause boundary layer [*Miura*, 1984; *Chen and Kivelson*, 1993], during the linear phase prior to rollup. Multiple current systems are generated by KHIs [*Masters et al.*, 2010], which may provide the needed energization source to create the high charge‐state ions that can produce X‐rays.

At Earth, *Taylor et al.* [2012] reported a dawn‐dusk asymmetry in rolled‐up vortices detection, with higher frequency on the postnoon dusk flank, while a previous study by *Hasegawa et al.* [2006] reported as many KHIs on either flank. Unlike Earth, the Jovian magnetosphere is populated by highly corotating plasma [*Thomsen et al.*, 2010; *Mauk et al.*, 2009], which contributes to a larger shear at the dawnside, where the corotation is sunward [*Johnson et al.*, 2014]. As a result, this larger shear is expected to favor the generation of KHI on the dawnside rather than on the duskside [*Desroche et al.*, 2012, 2013]. However, based on the development timescale of Kelvin‐Helmholtz vortices in relevance to Jovian orbital period, the structures at the dawn and dusk flanks may primarily originate from the same location [*Johnson et al.*, 2014], which could result in observation of rolled‐up vortices at earlier MLTs.

KHIs similar to those at Earth are less able to explain the first observation emission that originates closer to the nose of the magnetosphere, near to noon MLT. *Cowley et al.* [2007], however, find that flow shear along the open‐closed field line boundary would be important at Jupiter and capable of generating high‐latitude aurora. The shear increases when the magnetosphere is compressed due to increased angular velocity of the magnetospheric plasma, which could cause auroral emission to brighten [*Nichols et al.*, 2009], so it may be that flow shear is also relevant close to the nose.

It remains unclear as to why the hot spot feature is localized in these and previous observations [*Elsner et al.*, 2005; *Gladstone et al.*, 2002; *Branduardi‐Raymont et al.*, 2008] and restricted to limited longitudes of the Jovian pole. If the hot spot is driven by KHIs or dayside reconnection, then this may imply either that these processes are localized for the Jovian magnetosphere or that the high‐energy downward current region that produces X‐rays is localized.

The high‐energy electrons that generate the bremsstrahlung emission originate on the opposite side of the planet to the ion emission, in regions between 02:00 and 06:30 magnetospheric local time. At Earth, similar features are associated with whistler mode waves and the dawn chorus. The possible periodicity in the 5–10 min range may be consistent with this explanation. Dawn storms at Jupiter have been observed in the UV on several prior occasions [e.g., *Gustin et al.*, 2006; *Clarke et al.*, 2009; *Nichols et al.*, 2009] and may be capable of supplying sufficiently energetic electrons for X‐ray bremsstrahlung emission. The hard X‐ray emission from high‐energy electron precipitation also increased during the first observation. Brightening of the UV main emission has been observed to occur coincident with solar wind shocks [e.g., *Nichols et al.*, 2009]. Simultaneous UV‐X‐ray observations would help to further constrain these connections between brightness variation in the UV main oval and increased hard X‐ray emission from high‐energy electrons in this region. They would also help to identify global current systems, with UV helping to highlight upward currents (away from the planet) and X‐rays from ions helping to identify downward currents (toward the planet).

### 10.2 The Auroral Enhancement

#### 10.2.1 Where Is the Auroral Enhancement Source?

In the quadrant from 180° to 270° S3 longitude, we note the largest change in auroral emission between the two observations the bright auroral enhancement on day of year 276.25. The brightest peak of this event lasts ∼20 min, 2–4 times longer than the flare reported by *Elsner et al.* [2005].

Figures 7 and 14 show that the ion emission originates from a range of different latitudes and therefore maps to several different closed and open field line regions, suggesting that at this time, there may be several downward current regions on which the ions can precipitate. The precipitating particles also originate from a range of different magnetospheric local times across the dayside of Jupiter from dawn to close to dusk.

#### 10.2.2 What Process Connected to the ICME Drives the Observed Auroral Enhancement?

The auroral enhancement occurs 1–1.5 h prior to a bright non‐Io decametric radio burst (Figure 2), which has previously been found to relate to the impingement of a solar wind forward shock [*Gurnett et al.*, 2002; *Lamy et al.*, 2012; *Hess et al.*, 2012, 2014]. The mSWiM propagation also suggests the arrival of an ICME close to this time. The combination of this radio emission and the mSWiM‐predicted solar wind density peak leads us to believe that the bright X‐ray auroral enhancement is driven directly by this ICME.

What process could be directly responsible for this X‐ray brightening? The driver does not seem to be a continuation of the same process that produces the hot spot emission because the properties of the two emissions differ. The prominent differences between the AEQ and HSQ emission include a different population of precipitating particles (Figures 6 and 8); the enhancement emission is spatially less localized than the hot spot emission (Figures 3, 7, 8, and 10, 11, 12, 13, 14); the enhancement emission seems to increase temporally into a concentrated flare‐like event, with no significant periodicity in the ion emission (Figures 5 and 15), while the hot spot emission exhibits clear pulsations.

The AEQ features also seem atypical when compared with other X‐ray observations [*Elsner et al.*, 2005; *Gladstone et al.*, 2002; *Branduardi‐Raymont et al.*, 2008]. While the hot spot may be driven by KHIs or pulses of dayside reconnection close to a downward current region, we suggest that the auroral enhancement is driven by a less common process that is directly associated with the changing solar wind parameters induced by the ICME. Inspecting the mSWiM propagation (Figure 1) implies that the driver relates to either increased solar wind density or changing interplanetary magnetic field angle (as suggested by the rotation in *B*_*T*_). We propose two possible drivers based on these changing solar wind parameters, but note that they might not be independent drivers: (1) an ICME‐induced compression event or (2) an ICME‐induced instance of large‐scale dayside reconnection.

Increased ram pressure from the heightened solar wind density (Figure 1a) could drive a Jovian magnetosphere compression. The *Vogt et al.* [2011] mapping shows X‐ray emission from several regions inside the magnetosphere, suggesting that the ICME transfers energy into the magnetosphere, so that ions are sufficiently energetic for X‐ray production. This also raises questions as to the location of the downward currents (on which the ions precipitate) at this time. Compression events have been suggested to drive changes in Jupiter's current system and therefore acceleration processes [*Cowley et al.*, 2007; *Cowley and Bunce*, 2003a, 2003b]. Adjustments to the location of downward currents, induced by the compression, may therefore explain the observed broad spatial spread of ion emission, which during the auroral enhancement is not restricted to the hot spot as it normally is.

Alternatively, or in combination with a compression, a large‐scale instance of dayside reconnection may explain the observations. *Desroche et al.* [2012] showed that dayside reconnection would be confined to local regions on the magnetopause for certain IMF orientations, but varying IMF angle could lead dayside reconnection to occur across a larger proportion of the magnetopause. *Masters* [2015] further shows for Saturn that changing IMF angle can lead to increased reconnection voltages and a larger spatial scale of magnetopause reconnection. This could result in increased injection of solar wind particles and energization of a larger region of the outer magnetosphere plasma, explaining the observations of the larger spatial scale of emission and the observed change in the precipitating population from the spectra. The inverse of this mechanism may also help to explain reduced emission from the hot spot for some observations, since a less favorable IMF angle would suppress reconnection and therefore emission from the hot spot. Further comparison of Jupiter X‐ray emission with upstream IMF measurements would help to investigate this relationship.

The *Vogt et al.* [2011] mapping also lends weight to the argument that solar wind‐magnetosphere coupling is at work during this interval. It is possible that the solar wind compression and/or possible associated dayside reconnection for favorable IMF direction can lead to an opening of magnetic flux on the dayside, and concurrent X‐ray flaring. *Cravens et al.* [2003], addressing charge exchange, show that X‐ray emissivity from solar wind particles depends on solar wind velocity and density, which is in line with our observation of increased emission. We also found that the magnetospheric mapping suggests an open field line origin for at least some of the emission. This is supported by similarities between the AEQ spectrum and cometary spectra, which are known to be produced by solar wind charge exchange (from direct solar wind precipitation). However, we are cautious to note that the complex configuration of the Jovian magnetosphere at this time may not be accurately represented by the *Vogt et al.* [2011] mapping model, so the magnetospheric mapping at this time may be less reliable.

The low frequency of such ICME events, relative to the timescales of X‐ray observations, may help to explain why these features have not been previously reported in the literature and why the second observation seems to have an AEQ that is again largely devoid of emission. We also note that such events may be confused with hot spot emission, if they occur at a time when the hot spot is in the observable quadrant, as opposed to this observation where the hot spot was rotating out of view when the auroral enhancement occurred.

While we suggest that the solar wind does drive several changes in Jupiter's X‐ray aurora, we note that the importance of the solar wind as a driver of magnetospheric dynamics and that the existence of Dungey cycle processes at Jupiter remains a subject of debate [*McComas and Bagenal*, 2007, 2008; *Cowley et al.*, 2008].

Given that our findings are based on only two observations with this type of analysis, application of this approach to other observations would help to determine whether these features persist, how and where they originate, and whether there are systematic trends between the X‐ray aurora and solar wind.

## 11 Conclusion

We report the first X‐ray observation that was planned to coincide with an ICME arrival at Jupiter and find evidence for ICME‐induced changes in the northern X‐ray aurora. We observe changes in the morphology, spectra, and periodicity of the emission at this time. We particularly find an auroral enhancement by a factor of 8, occurring 1–1.5 h before a bright burst of non‐Io decametric radio emission, often associated with the arrival of an ICME‐induced fast‐forward shock [*Hess et al.*, 2012, 2014; *Lamy et al.*, 2012] and at a time when solar wind propagation models indeed predict an ICME arrival.

We have used *Vogt et al.* [2011] magnetospheric mapping to identify the origin of the X‐ray emission. This mapping suggests that most auroral X‐ray emissions came from precipitating ions with origins beyond 60 *R*_*J*_ on both open and closed field lines. Spatial uncertainties and uncertainties as to whether the magnetosphere is compressed or expanded at this time inhibit us from quantifying from which side of the magnetopause the majority of emission originates. The region between 50 and 70 *R*_*J*_ is dominated by 200–500 eV emission, which we attribute to precipitating high charge‐state magnetospheric sulfur ions. At higher latitudes that map between 70 and 120 *R*_*J*_ and to open field lines, there is a mixture of precipitating high charge‐state carbon/sulfur and oxygen ions.

In the hot spot, these separate origins for ions of different species is supported by periodicity measurements. In the first observation we find a strong 26 min period associated with the carbon/sulfur (200–500 eV) emission, but not with the oxygen (500–800 eV) emission. We do, however, find a 12 min period at a low level of significance in both the oxygen and carbon/sulfur emission. When the two populations are combined, the 12 min period becomes significant. The periods of 12 and 26 min in the first observation are distinctly shorter than the 42 min period we detect in the second observation, which is close to the 45 min timescale found by *Gladstone et al.* [2002].

X‐ray emission is concentrated in regions near to open field lines. On the basis of the magnetospheric local time of the source and the origin close to the magnetopause, alongside the periodicities and heightened solar wind conditions, we suggest that pulses of dayside reconnection [*Bunce et al.*, 2004; *Desroche et al.*, 2012] near a magnetospheric downward current region could be driving the X‐ray hot spot emission. We also suggest that the spectral, spatial, and temporal differences between the hot spot emission and auroral enhancement emission imply that they are not created by a continuation of the same process. Instead, we suggest that the auroral enhancement is directly driven by the ICME through a compression event and/or a larger‐scale instance of dayside reconnection than that producing the hot spot emission.

Other mechanisms in the outer magnetosphere, near the magnetopause, such as KHIs, may also have an important role in transferring momentum and energy in our observations, given that the Dungey cycle may well be less important for Jupiter than Earth [*McComas and Bagenal*, 2007, 2008; *Delamere and Bagenal*, 2010; *Johnson et al.*, 2014].

We believe that the approach of applying *Vogt et al.* [2011] model mapping to energy‐binned, subsolar longitude‐binned X‐rays offers excellent possibilities for mapping the origins of the Jovian X‐ray aurora and thus better understanding the Jovian outer magnetosphere and the processes occurring close to the magnetopause. Similar analysis on new and archival X‐ray observations is required to determine whether the features observed in these observations persist and how they relate to systematic trends in solar wind conditions. Combining observations of this kind with the approach and arrival of the Juno spacecraft in 2016 will offer further opportunities to understand the processes governing Jovian auroral X‐rays.

## Supporting information



Figures S1–S10
