# Supplementary material for: The impact of an ICME on the Jovian X‐ray aurora
Source: J Geophys Res Space Phys. 2016 Mar 22;121(3):2274–307. doi: 10.1002/2015JA021888 (PMC5111422; doi:10.1002/2015JA021888)
Supplement: Supplementary file 1 — Figures S1–S10 [file JGRA-121-2274-s001.pdf]

JOURNAL OF GEOPHYSICAL RESEARCH, VOL. ???, XXXX, DOI:10.1002/,

Supporting Information for The Impact of an ICME on the Jovian X-ray Aurora

William R. Dunn,[1,2] Graziella Branduardi-Raymont,[1] Ronald F. Elsner,[3] Marissa F. Vogt,[4] Laurent Lamy,[5] Peter G. Ford,[6] Andrew J. Coates,[1,2] G. Randall Gladstone,[7] Caitriona M. Jackman,[8] Jonathan D. Nichols,[9] I. Jonathan Rae,[1] Ali Varsani,[1,11] T. Kimura,[10,12] Kenneth C. Hansen,[13] Jamie M. Jasinski[1,2,13]

Contents of this file

1. Solar X-ray Flares: GOES SXI and Jovian X-ray Lightcurve Comparison
2. Equator Spectra
3. Spectral Extraction Regions for Jovian North Pole
4. Periodicities
5. Upstream Solar Wind Measurements From Pioneer 11, Voyager 1 and Voyager 2

S1: Solar X-ray Flares: GOES SXI and Jovian X-ray Lightcurve Comparison

During the Chandra X-ray observations reported in the article several solar flares occurred. These solar flare events are characterised by increased X-ray photon flux from the Sun, which leads to increased Jovian disk emission [Bhardwaj et al., 2005; 2006; Branduardi-Raymont et al., 2007B] because more X-rays are fluoresced and scattered in Jupiter's upper atmosphere.

In the article we demonstrate that the sulfur/carbon and oxygen emissions are concentrated into the aurora, showing that changes in solar photon flux are not responsible for the auroral changes we observe. If they were related, then we would not expect such distinct regional concentrations. In the supporting material, we further confirm that the lightcurve peak in the auroral enhancement quadrant (main article: figure 5) observed during the first observation, at DoY 276.25 (main article: section 4), was unrelated to solar flares. By doing this, we provide further evidence for the correlation between Jupiter's disk (and particularly equatorial) X-ray emission and solar emission [Bhardwaj et al., 2005; 2006; Branduardi Raymont et al., 2007B].

Supporting Information (SI) Figure 1 shows the Sun's X-ray flux as recorded by the Solar X-ray Imager (SXI) on the GOES spacecraft throughout the two Chandra X-ray observations of Jupiter discussed in the main text. The black arrows plotted onto the figures indicate the times of the two Chandra observations, shifted to account for light travel time. SI Figure 1 shows that the X-ray flux of the Sun peaked three times during the first Chandra observation. The first two X-ray flux peaks correspond to an order of magnitude increase in Solar X-ray flux and the third peak contributes to a half order of magnitude increase. During the final hour of the second Chandra observation, there is only one half order of magnitude peak in the solar flux.

SI Figure 2 shows the X-ray lightcurves for the equatorial zone on Jupiter (using latitudes: -60° - 60°) for each of the two observations. The equatorial lightcurve for the first observation features peaks, at the times when the flares observed by GOES SXI are expected to arrive at Jupiter. This shows that the disk emission replicates the GOES SXI data well, in support of the conclusions of Bhardwaj et al. [2005; 2006] and Branduardi-Raymont et al. [2007B] that the Jovian X-ray disk emission is generated by scattered and fluorescing solar photons.

The equatorial lightcurves (SI Figure 2), which match the GOES SXI data well, feature very different morphology to the auroral lightcurves (SI Figure 3), which do not match the GOES SXI data. While the equatorial lightcurves (SI Figure 2) peak at the light-travel shifted times of the GOES lightcurves (SI Figure 1), the auroral lightcurve (SI Figure 3) do not vary with the first flare in a notable way. Unfortunately, at the time when the second solar flare arrived at Jupiter, the hot spot rotates into view on the disk. Disentangling the X-ray emission from the second solar flare from the increase in emission because of the hot spot visibility is challenging. However, the auroral zone associated with the hot spot remains bright for more than an hour after the solar flare has dimmed from the equatorial zone. This further supports an auroral origin for the majority of the additional emission observed in this region throughout the first observation. The solar flares may contribute a small fraction of the auroral emission, which may explain the appearance of common solar Fe XXI, Ne X and Mg XI lines in the hot spot spectrum (Main Article section 5).

There is no solar flare between DoY 276.2 - 276.4 and the equator has dimmed to a normal level by this point, meaning that solar flares are not responsible for Jupiter's auroral enhancement feature, which we suggest is caused by the ICME. For the second observation, the only solar flare

[1]Mullard Space Science Laboratory, Department of Space & Climate Physics, University College London, Holmbury St. Mary, Dorking, Surrey RH5 6NT, UK
[2]The Centre for Planetary Science at UCL/Birkbeck, Gower Street, London, WC1E 6BT, UK
[3]ZP12, NASA Marshall Space Flight Center, USA
[4]Center for Space Physics, Boston University, USA
[5]LESIA, Observatoire de Paris, CNRS, UPMC, Université Paris Diderot, Meudon, France
[6]Kavli Institute for Astrophysics and Space Research, MIT, Cambridge MA, USA
[7]Space Science & Engineering Division, Southwest Research Institute, San Antonio, Texas, USA
[8]Department of Physics & Astronomy, University of Southampton, Southampton SO17 1BJ, UK
[9]Department of Physics & Astronomy, University of Leicester, Leicester UK
[10]Institute of Space and Astronautical Science, Japan Aerospace Exploration Agency, Sagamihara, Japan.
[11]Space Research Institute, Austrian Academy of Sciences, Graz, Austria
[12]Nishina Center for Accelerator-Based Science, RIKEN, 2-1, Hirosawa, Wako, Saitama, Japan
[13]Department of Atmospheric, Oceanic and Space Sciences, University of Michigan, Ann Arbor, Michigan, USA

Copyright 2016 by the American Geophysical Union.
0148-0227/16/$9.00

occurs at the end of the observation and its impact on the equatorial lightcurve can start to be seen by a factor 2 increase in emission in the final hour of the observation. This appears to have minimal impact on the Northern Auroral zone lightcurve, again implying that the solar flares are not the main contributor to the variations we observe in the auroral emission.

SI Figure 4 highlights the impact of the solar flares on Jupiter's disk spectrum in the first observation in comparison with the non-solar flare spectrum of the second observation.

S2: Spectral Extraction

SI Figure 5 shows the regions utilised for spectra extraction. The Northern aurora spectra was extracted from the region enclosed by the overlap between the upper square and the circle. Any emission not inside the region where these two shapes overlap was ignored. The equatorial spectra was extracted from the region of overlap between the middle square and the circle. Again, emission which was not inside of both shapes was ignored.

S3: Periodicities

SI Figure 6 shows the events (in red) included in generating the PSDs from the hot spot region for the first (left) and second (right) observations respectively. The periodicities were apparent when the PSD was generated for all X-rays above 60° latitude in the quadrant, but the periods became more significant when the region selected was honed onto the hot spot.

SI Figure 7 demonstrates that no periodicity could be detected in the Auroral Enhancement Quadrant. We sampled a range of smaller regions across this quadrant from low to high latitude and at varying longitudes and were still unable to detect a significant period in this region. Logistically, the periodicity may exist, but the paucity of events in any given region makes it difficult to detect. It also may be that the flare-like auroral enhancement complicates this by 'washing-out' any period. Additionally, we note that periodicity in the hot spot is a transient feature (e.g: Branduardi-Raymont et al. 2004; 2007A; Elsner et al. 2005 did not detect any strict periodicity). If the driver for the enhancement is a compression then this would be a different driver to a localised instance of reconnection. Alternatively, if it is a large-scale reconnection event, then this would stretch across a larger region of the magnetopause, providing a broader range of planet-magnetopause transit times.

For the hard X-rays (SI Figure 8), from precipitating electrons, there is a suggestion of possible periodicity on 5-10 minute timescales with a PCO of 10^{-4}-10^{-3}. For other discrete energies there was no detectable statistically significant periodicity.

S4: Upstream Solar Wind Measurements From Pioneer 11, Voyager 1 and Voyager 2

SI Figure 9 and 10 show distributions of solar wind density and velocity respectively from the Pioneer 11, Voyager 1 and Voyager 2 spacecrafts. The datasets are based on 60-minute resolution measurements, made when the spacecraft were beyond 4 AU and upstream of Jupiter sampling the solar wind. The frequency plots represent the raw number of data points. From these, medians and means are used for comparison with the ICME conditions in section 2 of the article.

Data S1: Supporting Information Figures

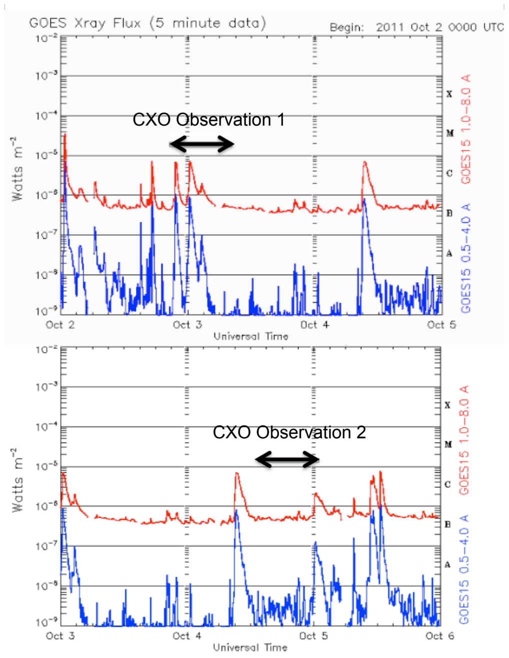

Figure 1. 5-minute binned X-ray flux from the Sun as recorded by the Solar X-ray Imager on the GOES spacecraft throughout the time period of our first (upper plot) and second (lower plot) Chandra X-ray observations. The black arrows indicate the timing of the respective X-ray observations and account for the GOES-Jupiter-Earth light travel time for solar X-rays fluoresced and scattered from Jupiter's disk.

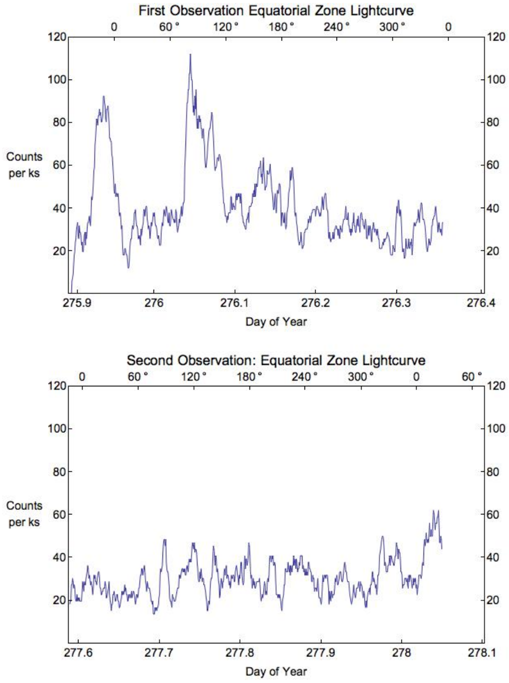

Figure 2. X-ray Equator Lightcurves with one minute binning for the first (upper) and second (lower) observations. The equatorial lightcurves use events between S3 latitudes of -60° and 60°. The lightcurve morphology appears to be a good a match for the GOES lightcurve in both cases.

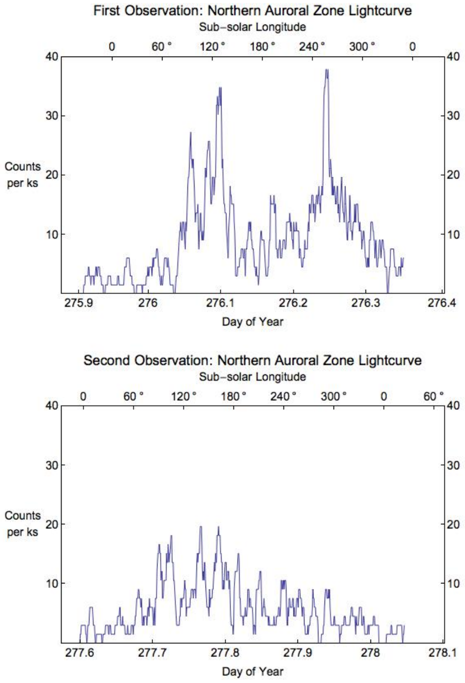

Figure 3. X-ray Aurora Lightcurves with one minute binning for the first (upper) and second (lower) observations. The auroral lightcurves use events above S3 latitudes of 60°. The lightcurve morphology is very unlike that of the equator or GOES lightcurves.

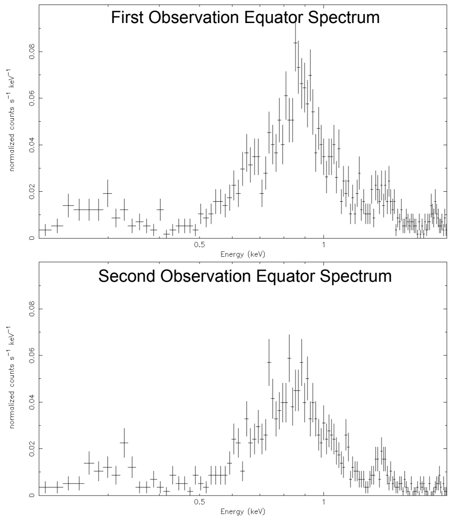

Figure 4. Spectra from Jupiter's Equator for the first (upper) and second (lower) observations. The change in the first observation spectrum from the solar flare is clearly identifiable.

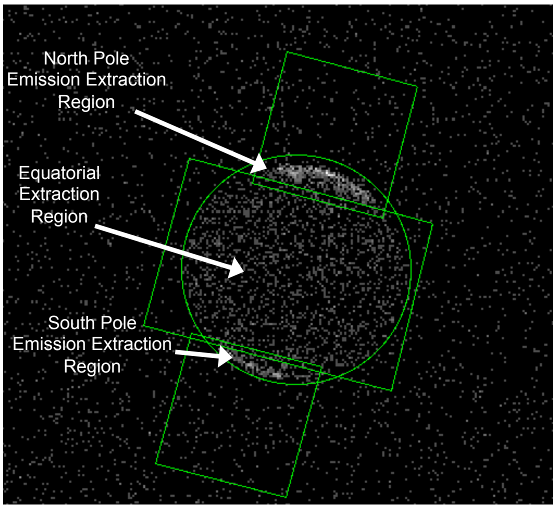

Figure 5. Regions used for spectra extraction. The northern aurora spectrum was extracted from the region of overlap between the top square and the circle. No emission outside of the combination of these two was included. The equatorial spectrum was extracted from the region of overlap between the middle rectangle and the circle. No emission outside of this overlap was included.

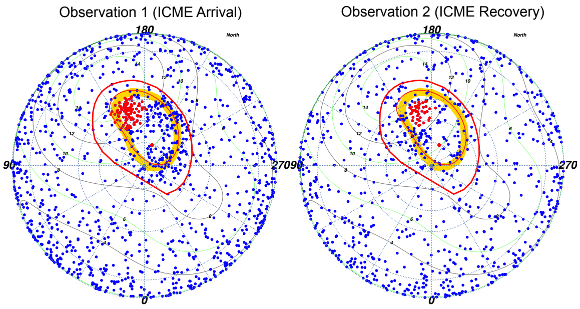

Figure 6. Polar projections indicating the events (red) which were used to generate the PSDs for the hot spot discussed in section 8 of the main text.

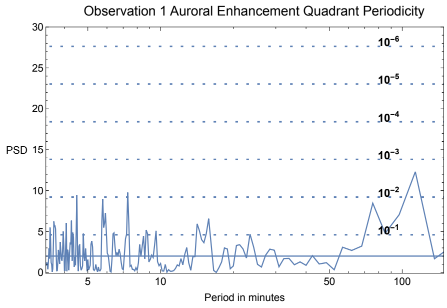

Figure 7. PSD generated from the events in the Auroral Enhancement Quadrant (Latitude: Above 60° Longitude: 180°-270°). No significant period is present.

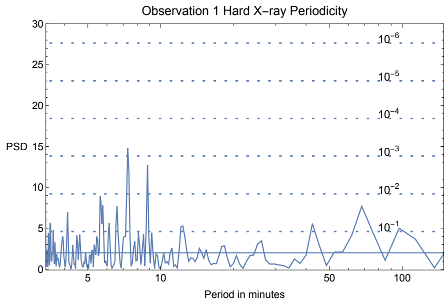

Figure 8. PSD generated from the hard X-ray events in the Auroral Enhancement Quadrant (Latitude: Above 60° Longitude: 180°-270°). There are hints at a 5-10 minute period.

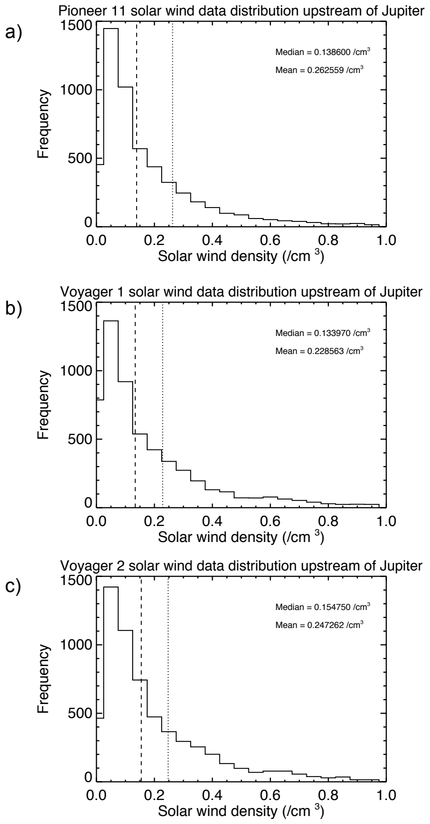

Figure 9. Distributions of solar wind density measured upstream from Jupiter by a) Pioneer 11 b) Voyager 1 and c) Voyager 2 spacecrafts.

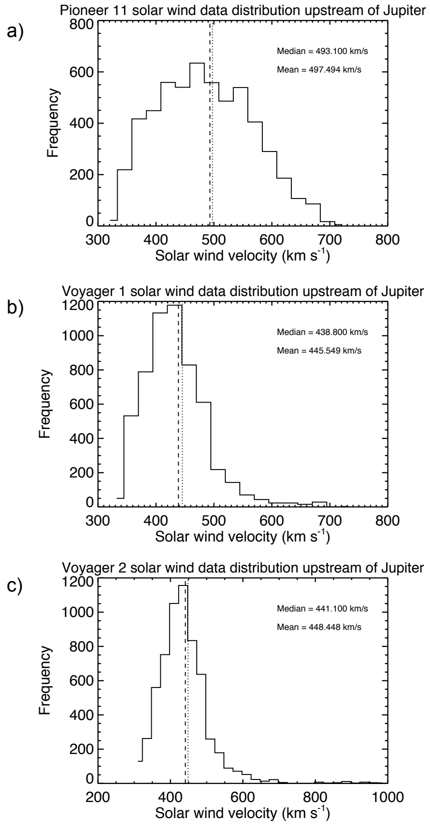

Figure 10. Distributions of solar wind velocity measured upstream from Jupiter by a) Pioneer 11 b) Voyager 1 and c) Voyager 2 spacecrafts.
